# Worst Singularities of Plane Curves of Given Degree

**DOI:** 10.1007/s12220-017-9762-y

**Published:** 2017-02-07

**Authors:** Ivan Cheltsov

**Affiliations:** 1 School of Mathematics, University of Edinburgh, Peter Guthrie Tait Road, Edinburgh, EH9 3FD UK; 2 Laboratory of Algebraic Geometry, GU-HSE, 6 Usacheva street, Moscow, Russia 119048

**Keywords:** Log canonical threshold, Plane curve, GIT-stability, $\alpha$-Invariant of Tian, Smooth surface, 14H20, 14H50, 14J70 (primary), 14E05, 14L24, 32Q20 (secondary)

## Abstract

We prove that $\frac{2}{d}, \frac{2d-3}{(d-1)^2}, \frac{2d-1}{d(d-1)}, \frac{2d-5}{d^2-3d+1}$ and $\frac{2d-3}{d(d-2)}$ are the smallest log canonical thresholds of reduced plane curves of degree $d\geqslant 3$, and we describe reduced plane curves of degree *d* whose log canonical thresholds are these numbers. As an application, we prove that $\frac{2}{d}, \frac{2d-3}{(d-1)^2}, \frac{2d-1}{d(d-1)}, \frac{2d-5}{d^2-3d+1}$ and $\frac{2d-3}{d(d-2)}$ are the smallest values of the $\alpha$-invariant of Tian of smooth surfaces in ${\mathbb {P}}^3$ of degree $d\geqslant 3$. We also prove that every reduced plane curve of degree $d\geqslant 4$ whose log canonical threshold is smaller than $\frac{5}{2d}$ is GIT-unstable for the action of the group $\mathrm {PGL}_3({\mathbb {C}})$, and we describe GIT-semistable reduced plane curves with log canonical thresholds $\frac{5}{2d}$.

## Introduction

Let $C_d$ be a *reduced* plane curve in ${\mathbb {P}}^2$ of degree $d\geqslant 3$, and let *P* be a point in $C_d$. The curve $C_d$ can have *any* given plane curve singularity at *P* provided that its degree *d* is *sufficiently* big. Thus, it is natural to ask

### Question 1.1

What is the *worst* singularity that $C_d$ can have at *P*?

Denote by $m_P$ the multiplicity of the curve $C_d$ at the point *P*, and denote by $\mu (P)$ the Milnor number of the point *P*. If we use $m_P$ to measure the singularity of $C_d$ at the point *P*, then a union of *d* lines passing through *P* is an answer to Question 1.1, since $m_P\leqslant d$, and $m_P=d$ if and only if $C_d$ is a union of *d* lines passing through *P*. If we use the Milnor number $\mu (P)$, then the answer would be the same, since $\mu (P)\leqslant (d-1)^2$, and $\mu (P)=(d-1)^2$ if and only if $C_d$ is a union of *d* lines passing through *P*. Alternatively, we can use the number

$$\begin{aligned} \mathrm {lct}_P\big ({\mathbb {P}}^2,C_d\big )=\mathrm {sup}\Big \{\lambda \in {\mathbb {Q}}\ \Big |\ \text {the log pair}\ \big ({\mathbb {P}}^2, \lambda C_d\big )\ \text {is log canonical at }P\Big \}, \end{aligned}$$

which is known as the *log canonical threshold* of the log pair $({\mathbb {P}}^2, C_d)$ at the point *P* or the log canonical threshold of the curve $C_d$ at the point *P* (see [4, Definition 6.34]). The smallest $\mathrm {lct}_P({\mathbb {P}}^2,C_d)$ when *P* runs through all points in $C_d$ is usually denoted by $\mathrm {lct}({\mathbb {P}}^2,C_d)$. Note that

$$\begin{aligned} \frac{1}{m_P}\leqslant \mathrm {lct}_P\big ({\mathbb {P}}^2,C_d\big )\leqslant \frac{2}{m_P}. \end{aligned}$$

This is well known (see, [4, Exercise 6.18] and [4, Lemma 6.35]). So, the smaller $\mathrm {lct}_P({\mathbb {P}}^2,C_d)$, the worse singularity of the curve $C_d$ at the point *P* is.

### Example 1.2

Suppose that $C_d$ is given by $x_1^{n_1}x_2^{n_2}(x_1^{m_1}+x_2^{m_2})=0$ up to analytic change of local coordinates, where $m_1$ and $m_2$ are positive integers, and $n_1,n_2\in \{0,1\}$. Then

$$\begin{aligned} \mathrm {lct}_P\big ({\mathbb {P}}^2,C_d\big )=\mathrm {min}\Bigg \{1,\frac{\frac{1}{m_1}+\frac{1}{m_2}}{1+\frac{n_1}{m_1}+\frac{n_2}{m_2}}\Bigg \} \end{aligned}$$

by [8, Proposition 2.2].

Log canonical thresholds of plane curves have been intensively studied (see, for example, [8]). Surprisingly, they give the same answer to Question 1.1 by

### Theorem 1.3

([1, Theorem 4.1]) One has $\mathrm {lct}_P({\mathbb {P}}^2,C_d)\geqslant \frac{2}{d}$. Moreover, $\mathrm {lct}({\mathbb {P}}^2,C_d)=\frac{2}{d}$ if and only if $C_d$ is a union of *d* lines that pass through *P*.

In this paper we want to address

### Question 1.4

What is the *second worst* singularity that $C_d$ can have at *P*?

To give a *reasonable* answer to this question, we have to disregard $m_P$ by obvious reasons. Thus, we will use the numbers $\mu (P)$ and $\mathrm {lct}_P({\mathbb {P}}^2,{\mathbb {C}}_d)$. For cubic curves, they give the same answer.

### Example 1.5

Suppose that $d=3, m_P<3$ and *P* is a singular point of $C_3$. Then *P* is a singular point of type ${\mathbb {A}}_1, {\mathbb {A}}_2$ or ${\mathbb {A}}_3$. Moreover, if $C_3$ has singularity of type ${\mathbb {A}}_3$ at *P*, then $C_3=L+C_2$, where $C_2$ is a smooth conic, and *L* is a line tangent to $C_2$ at *P*. Furthermore, we have

$$\begin{aligned} \mu (P)=\left\{ \begin{aligned}&1\ \text {if }C_3\text { has }{\mathbb {A}}_1\text { singularity at }P,\\&2\ \text {if }C_3\text { has }{\mathbb {A}}_2\text { singularity at }P,\\&3\ \text {if }C_3\text { has }{\mathbb {A}}_3\text { singularity at }P.\\ \end{aligned}\right. \end{aligned}$$

Similarly, we have

$$\begin{aligned} \mathrm {lct}_P\big ({\mathbb {P}}^2,C_3\big )=\left\{ \begin{aligned}&1\ \text {if }C_3\text { has }{\mathbb {A}}_1\text { singularity at }P,\\&\frac{5}{6}\ \text {if }C_3\text { has }{\mathbb {A}}_2\text { singularity at }P,\\&\frac{3}{4}\ \text {if }C_3\text { has }{\mathbb {A}}_3\text { singularity at }P.\\ \end{aligned}\right. \end{aligned}$$

For quartic curves, the numbers $\mu (P)$ and $\mathrm {lct}_P({\mathbb {P}}^2,{\mathbb {C}}_d)$ give different answers to Question 1.4.

### Example 1.6

Suppose that $d=4, m_P<4$ and *P* is a singular point of $C_4$. Going through the list of all possible singularities that $C_P$ can have at *P* (see, for example, [6]), we obtain

$$\begin{aligned} \mu (P)=\left\{ \begin{aligned}&6\ \text {if }C_4\text { has }{\mathbb {D}}_6\text { singularity at }P,\\&6\ \text {if }C_4\text { has }{\mathbb {A}}_6\text { singularity at }P,\\&6\ \text {if }C_4\text { has }{\mathbb {E}}_6\text { singularity at }P,\\&7\ \text {if }C_4\text { has }{\mathbb {A}}_7\text { singularity at }P,\\&7\ \text {if }C_4\text { has }{\mathbb {E}}_7\text { singularity at }P,\\ \end{aligned}\right. \end{aligned}$$

and $\mu (P)<6$ in all remaining cases. Similarly, we get

$$\begin{aligned} \mathrm {lct}_P\big ({\mathbb {P}}^2,C_4\big )=\left\{ \begin{aligned}&\frac{5}{8}\ \text {if }C_4\text { has }{\mathbb {A}}_7\text { singularity at }P,\\&\frac{5}{8}\ \text {if }C_4\text { has }{\mathbb {D}}_5\text { singularity at }P,\\&\frac{3}{5}\ \text {if }C_4\text { has }{\mathbb {D}}_6\text { singularity at }P,\\&\frac{7}{12}\ \text {if }C_4\text { has }{\mathbb {E}}_6\text { singularity at }P,\\&\frac{5}{9}\ \text {if }C_4\text { has }{\mathbb {E}}_7\text { singularity at }P,\\ \end{aligned}\right. \end{aligned}$$

and $\mathrm {lct}_P({\mathbb {P}}^2,C_4)>\frac{5}{8}$ in all remaining cases.

Recently, Arkadiusz Płoski proved that $\mu (P)\leqslant (d-1)^2-\lfloor \frac{d}{2}\rfloor$ provided that $m_P<d$. Moreover, he described $C_d$ in the case when $\mu (P)=(d-1)^2-\lfloor \frac{d}{2}\rfloor$. To present his description, we need

### Definition 1.7

The curve $C_d$ is an *even Płoski* curve if *d* is even, the curve $C_d$ has $\frac{d}{2}\geqslant 2$ irreducible components that are smooth conics passing through *P*, and all irreducible components of $C_d$ intersect each other pairwise at *P* with multiplicity 4. The curve $C_d$ is an *odd Płoski* curve if *d* is odd, the curve $C_d$ has $\frac{d+1}{2}\geqslant 2$ irreducible components that all pass through $P, \frac{d-1}{2}$ irreducible component of the curve $C_d$ are smooth conics that intersect each other pairwise at *P* with multiplicity 4, and the remaining irreducible component is a line in ${\mathbb {P}}^2$ that is tangent at *P* to all other irreducible components. We say that $C_d$ is *Płoski* curve if it is either an even Płoski curve or an odd Płoski curve.

Each Płoski curve has unique singular point. If $d=4$, then $C_4$ is a Płoski curve if and only if it has a singular point of type ${\mathbb {A}}_7$. Thus, if $d=4$, then $\mu (P)=(d-1)^2-\lfloor \frac{d}{2}\rfloor =7$ if and only if either $C_4$ is a Płoski curve and *P* is its singular point or $C_4$ has singularity ${\mathbb {E}}_7$ at the point *P* (see Example 1.6). For $d\geqslant 5$, Płoski proved

### Theorem 1.8

([10, Theorem 1.4]) If $d\geqslant 5$, then $\mu (P)=(d-1)^2-\lfloor \frac{d}{2}\rfloor$ if and only if $C_d$ is a Płoski curve and *P* is its singular point.

This result gives a *very good* answer to Question 1.4. The main goal of this paper is to give an answer to Question 1.4. using log canonical thresholds. Namely, we will prove that

$$\begin{aligned} \mathrm {lct}_P\big ({\mathbb {P}}^2,C_d\big )\geqslant \frac{2d-3}{(d-1)^2} \end{aligned}$$

provided that $m_P<d$, and we will describe $C_d$ in the case when $\mathrm {lct}_P({\mathbb {P}}^2,C_d)=\frac{2d-3}{(d-1)^2}$. To present this description, we need

### Definition 1.9

The curve $C_d$ has singularity of type ${\mathbb {T}}_r$ (resp., ${\mathbb {K}}_r, \widetilde{{\mathbb {T}}}_{r}, \widetilde{{\mathbb {K}}}_{r}$) at the point *P* if the curve $C_d$ can be given by $x_1^r=x_1x_2^r$ (resp., $x_1^{r}=x_2^{r+1}, x_2x_1^{r-1}=x_1x_2^{r}, x_2x_1^{r-1}=x_2^{r+1}$) up to analytic change of coordinates at the point *P*.

Note that ${\mathbb {T}}_2={\mathbb {A}}_3, {\mathbb {K}}_2={\mathbb {A}}_2, \widetilde{{\mathbb {T}}}_{2}=\widetilde{{\mathbb {K}}}_{2}={\mathbb {A}}_1, \widetilde{{\mathbb {K}}}_{3}={\mathbb {D}}_5, \widetilde{{\mathbb {T}}}_{3}={\mathbb {D}}_6, {\mathbb {K}}_3={\mathbb {E}}_6$ and ${\mathbb {T}}_3={\mathbb {E}}_7$. Furthermore, since we assume that $d\geqslant 3$, the formula in Example 1.2 gives

$$\begin{aligned} \mathrm {lct}_P\big ({\mathbb {P}}^2,C_d\big )=\left\{ \begin{aligned}&\frac{2d-3}{(d-1)^2}\ \text {if }C_d\text { has }{\mathbb {T}}_{d-1} \text {singularity at }P,\\&\frac{2d-1}{d(d-1)}\ \text {if }C_d\text { has }{\mathbb {K}}_{d-1} \text {singularity at }P,\\&\frac{2d-5}{d^2-3d+1}\ \text {if }C_d\text { has } \widetilde{{\mathbb {T}}}_{d-1}\text { singularity at }P,\\&\frac{2d-3}{d(d-2)}\ \text {if }C\text { has } \widetilde{{\mathbb {K}}}_{d-1}\text { singularity at }P,\\ \end{aligned}\right. \end{aligned}$$

where $\frac{2}{d}<\frac{2d-3}{(d-1)^2}<\frac{2d-1}{d(d-1)}<\frac{2d-5}{d^2-3d+1}\leqslant \frac{2d-3}{d(d-2)}$. In this paper we will prove

### Theorem 1.10

Suppose that $d\geqslant 4$ and $\mathrm {lct}_P({\mathbb {P}}^2,C_d)\leqslant \frac{2d-3}{d(d-2)}$. Then one of the following holds:

1. $$m_P=d$$,
2. the curve $C_d$ has singularity of type ${\mathbb {T}}_{d-1}, {\mathbb {K}}_{d-1}, \widetilde{{\mathbb {T}}}_{d-1}$ or $\widetilde{{\mathbb {K}}}_{d-1}$ at the point *P*,
3. $d=4$ and $C_d$ is a Płoski quartic curve (in this case $\mathrm {lct}_P({\mathbb {P}}^2,C_d)=\frac{5}{8})$.

This result describes the *five worst* singularities that $C_d$ can have at the point *P*. In particular, Theorem 1.10 answers Question 1.4. This answer is very different from the answer given by Theorem 1.8. Indeed, if $C_d$ is a Płoski curve, $d>3$ and *P* is its singular point, then

$$\begin{aligned} \mathrm {lct}_P\big ({\mathbb {P}}^2,C_d\big )=\frac{5}{2d}>\frac{2d-3}{(d-1)^2}. \end{aligned}$$

The proof of Theorem 1.10 implies one result that is interesting on its own. To describe it, let us identify the curve $C_d$ with a point in the space $|{\mathcal {O}}_{{\mathbb {P}}^2}(d)|$ that parameterizes all (not necessarily reduced) plane curves of degree *d*. Since the group $\mathrm {PGL}_3({\mathbb {C}})$ acts on $|{\mathcal {O}}_{{\mathbb {P}}^2}(d)|$, it is natural to ask whether $C_d$ is GIT-stable (resp., GIT-semistable) for this action or not. For small *d*, its answer is classical and immediately follows from the Hilbert–Mumford criterion (see [9, Chapter 2.1]).

### Example 1.11

([9, Chapter 4.2]) If $d=3$, then $C_3$ is GIT-stable (resp., GIT-semistable) if and only if $C_3$ is smooth (resp., $C_3$ has at most ${\mathbb {A}}_1$ singularities). If $d=4$, then $C_4$ is GIT-stable (resp., GIT-semistable) if and only if $C_4$ has at most ${\mathbb {A}}_1$ and ${\mathbb {A}}_2$ singularities (resp., $C_4$ has at most singular double points and $C_4$ is not a union of a cubic with an inflectional tangent line).

Paul Hacking, Hosung Kim and Yongnam Lee noticed that the log canonical threshold $\mathrm {lct}({\mathbb {P}}^2,C_d)$ and GIT-stability of the curve $C_d$ are closely related. In particular, they proved

### Theorem 1.12

([5, Propositions 10.2 and 10.4], [7, Theorem 2.3]) If $\mathrm {lct}({\mathbb {P}}^2,C_d)\geqslant \frac{3}{d}$, then the curve $C_d$ is GIT-semistable. If $d\geqslant 4$ and $\mathrm {lct}({\mathbb {P}}^2,C_d)>\frac{3}{d}$, then the curve $C_d$ is GIT-stable.

This gives a *sufficient* condition for the curve $C_d$ to be GIT-stable (resp, GIT-semistable). However, this condition is not a *necessary* condition. Let us give two examples that illustrate this.

### Example 1.13

([13, p. 268], [5, Example 10.5]) Suppose that $d=5$, the quintic curve $C_5$ is given by

$$\begin{aligned} x^5+\Big (y^2-xz\Big )^2\Big (\frac{x}{4}+y+z\Big )=x^2\Big (y^2-xz\Big )\Big (x+2y\Big ), \end{aligned}$$

and $P=[0:0:1]$. Then $C_5$ is irreducible and has singularity ${\mathbb {A}}_{12}$ at the point *P*. In particular, it is rational. Furthermore, the curve $C_5$ is GIT-stable (see, for example, [9, Chapter 4.2]). On the other hand, it follows from Example 1.2 that

$$\begin{aligned} \mathrm {lct}\big ({\mathbb {P}}^2,C_5\big )=\mathrm {lct}_P\big ({\mathbb {P}}^2,C_5\big )=\frac{1}{2}+\frac{1}{13}=\frac{15}{26}<\frac{3}{5}. \end{aligned}$$

### Example 1.14

Suppose that $C_d$ is a Płoski curve. Let *P* be its singular point, and let *L* be a general line in ${\mathbb {P}}^2$. Then

$$\begin{aligned} \mathrm {lct}\big ({\mathbb {P}}^2,C_{d}+L\big )=\mathrm {lct}\big ({\mathbb {P}}^2,C_d\big )=\mathrm {lct}_P\big ({\mathbb {P}}^2,C_d\big )=\frac{5}{2d}<\frac{3}{d}. \end{aligned}$$

On the other hand, if *d* is even, then $C_d$ is GIT-semistable, and $C_d+L$ is GIT-stable. This follows from the Hilbert–Mumford criterion. Similarly, if *d* is odd, then $C_d$ is GIT-unstable, and $C_d+L$ is GIT-semistable.

In this paper we will prove the following result that complements Theorem 1.12.

### Theorem 1.15

If $\mathrm {lct}({\mathbb {P}}^2,C_d)<\frac{5}{2d}$, then $C_d$ is GIT-unstable. Moreover, if $\mathrm {lct}({\mathbb {P}}^2,C_d)\leqslant \frac{5}{2d}$, then $C_d$ is not GIT-stable. Furthermore, if $\mathrm {lct}({\mathbb {P}}^2,C_d)=\frac{5}{2d}$, then $C_d$ is GIT-semistable if and only if $C_d$ is an even Płoski curve.

Example 1.14 shows that this result is *sharp*. Surprisingly, its proof is very similar to the proof of Theorem 1.10. In fact, we will give a combined proof of both these theorems in Section 3.

In this paper we will also prove one application of Theorem 1.10. To describe it, we need

### Definition 1.16

([12, Appendix A], [3, Definition 1.20]) For a given smooth variety *V* equipped with an ample ${\mathbb {Q}}$-divisor $H_V$, let $\alpha _{V}^{H_{V}}:V\rightarrow {\mathbb {R}}_{\geqslant 0}$ be a function defined as

$$\begin{aligned} \alpha _{V}^{H_{V}}(O)=\mathrm {sup}\left\{ \lambda \in {\mathbb {Q}}\ \left| \begin{aligned}&\text {the pair}\ \left( V, \lambda D_V\right) \ \text {is log canonical at }O\\&\text {for every effective }{\mathbb {Q}}\text {-divisor}\ D_V\sim _{{\mathbb {Q}}} H_{V}\\ \end{aligned}\right. \right\} . \end{aligned}$$

Denote its infimum by $\alpha (V,H_V)$.

Let $S_d$ be a smooth surface in ${\mathbb {P}}^3$ of degree $d\geqslant 3$, let $H_{S_d}$ be its hyperplane section, let *O* be a point in $S_d$, and let $T_O$ be the hyperplane section of $S_d$ that is singular at *O*. Similar to $\mathrm {lct}_{P}({\mathbb {P}}^2,C_d)$, we can define

$$\begin{aligned} \mathrm {lct}_O\big (S_d,T_O\big )=\mathrm {sup}\Big \{\lambda \in {\mathbb {Q}}\ \Big |\ \text {the log pair}\ \big (S_d, \lambda T_O\big )\ \text {is log canonical at }O\Big \}. \end{aligned}$$

Then $\alpha _{S_d}^{H_{S_d}}(O)\leqslant \mathrm {lct}_{O}(S_d,T_O)$ by Definition 1.16. Note that $T_O$ is reduced, since the surface $S_d$ is smooth. In this paper we prove

### Theorem 1.17

If $$\alpha _{S_d}^{H_{S_d}}(O)<\frac{2d-3}{d(d-2)}$$, then

$$\begin{aligned} \alpha _{S_d}^{H_{S_d}}(O)=\mathrm {lct}_{O}\big (S_d, T_O\big )\in \Bigg \{\frac{2}{d}, \frac{2d-3}{(d-1)^2}, \frac{2d-1}{d(d-1)}, \frac{2d-5}{d^2-3d+1}\Bigg \}. \end{aligned}$$

Similarly, if $\alpha (S_d, H_{S_d})<\frac{2d-3}{d(d-2)}$, then

$$\begin{aligned} \alpha \big (S_d, H_{S_d}\big )=\inf _{O\in S_d}\Big \{\mathrm {lct}_{O}\big (S_d, T_O\big )\Big \}\in \Bigg \{\frac{2}{d}, \frac{2d-3}{(d-1)^2}, \frac{2d-1}{d(d-1)}, \frac{2d-5}{d^2-3d+1}\Bigg \}. \end{aligned}$$

If $d=3$, then we can drop the condition $\alpha _{S_d}^{H_{S_d}}(O)<\frac{2d-3}{d(d-2)}$ in Theorem 1.17, since $\frac{2d-3}{d(d-2)}=1$ in this case. Thus, Theorem 1.17 implies

### Corollary 1.18

([3, Corollary 1.24]) Suppose that $d=3$. Then $\alpha _{S_3}^{H_{S_3}}(O)=\mathrm {lct}_{O}(S_3, T_O)$.

If $d\geqslant 4$, we cannot drop the condition $\alpha _{S_d}^{H_{S_d}}(O)<\frac{2d-3}{d(d-2)}$ in Theorem 1.17 in general. Let us give two examples that illustrate this.

### Example 1.19

Suppose that $d=4$. Let $S_4$ be a quartic surface in ${\mathbb {P}}^3$ that is given by

$$\begin{aligned} t^3x+t^2yz+xyz(y+z)=0, \end{aligned}$$

and let *O* be the point [0 : 0 : 0 : 1]. Then $S_4$ is smooth, and $T_O$ has singularity ${\mathbb {A}}_1$ at *O*, which implies that $\mathrm {lct}_{O}(S_4, T_O)=1$. Let $L_y$ be the line $x=y=0$, let $L_z$ be the line $x=z=0$, and let $C_2$ be the conic $y+z=xt+yz=0$. Then $L_y, L_z$ and $C_2$ are contained in $S_4$, and $O=L_y\cap L_z\cap C_2$. Moreover,

$$\begin{aligned} L_y+L_z+\frac{1}{2}C_2\sim 2H_{S_4}, \end{aligned}$$

because the divisor $2L_y+2L_z+C_2$ is cut out on $S_4$ by $tx+yz=0$. Furthermore, the log pair $(S_4,L_y+L_z+\frac{1}{2}C_2)$ is not log canonical at *O*, so that $\alpha _{S_4}^{H_{S_4}}(O)<1$ by Definition 1.16.

### Example 1.20

Suppose that $d\geqslant 5$ and $T_O$ has ${\mathbb {A}}_1$ singularity at *O*. Then $\mathrm {lct}_{O}(S_d, T_O)=1$. Let $f:{\widetilde{S}}_d\rightarrow S_d$ be a blow up of the point *O*. Denote by *E* its exceptional curve. Then

$$\begin{aligned} \Big (f^*(H_{S_d})-\frac{11}{5}E\Big )^2=5-\frac{121}{25}>0. \end{aligned}$$

Hence, it follows from Riemann–Roch theorem there is an integer $n\geqslant 1$ such that the linear system $|f^*(5nH_{S_d})-11nE|$ is not empty. Pick a divisor ${\widetilde{D}}$ in this linear system, and denote by *D* its image on $S_d$. Then $(S_d,\frac{1}{5n}D)$ is not log canonical at *P*, since $\mathrm {mult}_{P}(D)\geqslant 11n$. On the other hand, $\frac{1}{5n}D\sim _{{\mathbb {Q}}} H_{S_d}$ by construction, so that $\alpha _{S_d}^{H_{_d}}(O)<1$ by Definition 1.16.

This work was carried out during the author’s stay at the Max Planck Institute for Mathematics in Bonn in 2014. We would like to thank the institute for the hospitality and very good working condition. We would like to thank Michael Wemyss for checking the singularities of the curve $C_{5}$ in Example 1.13. We would like to thank Alexandru Dimca, Yongnam Lee, Jihun Park, Hendrick Süß and Mikhail Zaidenberg for very useful comments.

## Preliminaries

In this section, we present results that will be used in the proof of Theorems 1.10, 1.15, 1.17. Let *S* be a smooth surface, let *D* be an effective non-zero ${\mathbb {Q}}$-divisor on the surface *S*, and let *P* be a point in the surface *S*. Write

$$\begin{aligned} D=\sum _{i=1}^{r}a_iC_i, \end{aligned}$$

where each $C_i$ is an irreducible curve on the surface *S*, and each $a_i$ is a non-negative rational number. Let us recall

### Definition 2.1

([4, § 6]) Let $\pi :{\widetilde{S}}\rightarrow S$ be a birational morphism such that ${\widetilde{S}}$ is smooth. Then $\pi$ is a composition of blow ups of smooth points. For each $C_i$, denote by ${\widetilde{C}}_i$ its proper transform on the surface ${\widetilde{S}}$. Let $F_1,\ldots , F_n$ be $\pi$-exceptional curves. Then

$$\begin{aligned} K_{{\widetilde{S}}}+\sum _{i=1}^{r}a_i{\widetilde{C}}_i+\sum _{j=1}^{n}b_jF_j\sim _{{\mathbb {Q}}}\pi ^{*}\big (K_{S}+D\big ) \end{aligned}$$

for some rational numbers $b_1,\ldots ,b_n$. Suppose, in addition, that $\sum _{i=1}^r{\widetilde{C}}_i+\sum _{j=1}^n F_j$ is a divisor with simple normal crossings. Then the log pair (*S*, *D*) is said to be *log canonical* at *P* if and only if the following two conditions are satisfied:

- $a_i\leqslant 1$ for every $C_i$ such that $P\in C_i$,
- $b_j\leqslant 1$ for every $F_j$ such that $\pi (F_j)=P$.

Similarly, the log pair (*S*, *D*) is said to be *Kawamata log terminal* at *P* if and only if $a_i<1$ for every $C_i$ such that $P\in C_i$, and $b_j<1$ for every $F_j$ such that $\pi (F_j)=P$.

Using just this definition, one can easily prove

### Lemma 2.2

Suppose that $r=3, P\in C_1\cap C_2\cap C_3$, the curves $C_1, C_2$ and $C_3$ are smooth at $P, a_1<1, a_2<1$ and $a_3<1$. Moreover, suppose that both curves $C_1$ and $C_2$ intersect the curve $C_3$ transversally at *P*. Furthermore, suppose that (*S*, *D*) is not Kawamata log terminal at *P*. Put $k=\mathrm {mult}_{P}(C_1\cdot C_2)$. Then $k(a_1+a_2)+a_3\geqslant k+1$.

### Proof

Put $S_0=S$ and consider a sequence of blow upswhere each $\pi _j$ is the blow up of the intersection point of the proper transforms of the curves $C_1$ and $C_2$ on the surface $S_{j-1}$ that dominates *P* (such point exists, since $k=\mathrm {mult}_{P}(C_1\cdot C_2)$). For each $\pi _j$, denote by $E_j^k$ the proper transform of its exceptional curve on $S_k$. For each $C_i$, denote by $C_i^k$ its proper transform on the surface $S_{k}$. Then

$$\begin{aligned} K_{S_{k}}+\sum _{i=1}^{n}a_iC_i^k+\sum _{j=1}^{k}\Big (j\big (a_1+a_2\big )+a_3-j\Big )E_j^k\sim _{{\mathbb {Q}}}(\pi _1\circ \pi _2\circ \cdots \circ \pi _k)^{*}\Big (K_{S}+D\Big ), \end{aligned}$$

and $\sum _{i=1}^{n}C_i^k+\sum _{j=1}^{k}E_j$ is a simple normal crossing divisor in every point of $\cup _{j=1}^{k}E_j$. Thus, it follows from Definition 2.1 that there exists $l\in \{1,\ldots ,k\}$ such that $l(a_1+a_2)+a_3\geqslant l+1$, because (*S*, *D*) is not Kawamata log terminal at *P*. If $l=k$, then we are done. So, we may assume that $l<k$. If $k(a_1+a_2)+a_3<k+1$, then $a_1+a_2<1+\frac{1}{k}-a_3\frac{1}{k}$, which implies that

$$\begin{aligned} l+1\leqslant & {} l\big (a_1+a_2\big )+a_3<\Bigg (l+\frac{l}{k}-a_3\frac{l}{k}\Bigg )+a_3=l+\frac{l}{k}+a_3\Bigg (1-\frac{l}{k}\Bigg )\\\leqslant & {} l+\frac{l}{k}+\Bigg (1-\frac{l}{k}\Bigg )=l+1, \end{aligned}$$

because $a_3<1$. Thus, the obtained contradiction shows that $k(a_1+a_2)+a_3\geqslant k+1$.

$$\square $$

### Corollary 2.3

Suppose that $r=2, P\in C_1\cap C_2$, the curves $C_1$ and $C_2$ are smooth at $P, a_1<1$ and $a_2<1$. Put $k=\mathrm {mult}_{P}(C_1\cdot C_2)$. If (*S*, *D*) is not Kawamata log terminal at *P*, then $k(a_1+a_2)\geqslant k+1$.

The log pair (*S*, *D*) is called *log canonical* if it is log canonical at every point of *S*. Similarly, the log pair (*S*, *D*) is called *Kawamata log terminal* if it is Kawamata log terminal at every point of the surface *S*.

### Remark 2.4

Let *R* be any effective ${\mathbb {Q}}$-divisor on *S* such that $R\sim _{{\mathbb {Q}}} D$ and $R\ne D$. Put

$$\begin{aligned} D_{\epsilon }=(1+\epsilon ) D-\epsilon R, \end{aligned}$$

where $\epsilon$ is a non-negative rational number. Then $D_{\epsilon }\sim _{{\mathbb {Q}}} D$. Moreover, since $R\ne D$, there exists the greatest rational number $\epsilon _0\geqslant 0$ such that the divisor $D_{\epsilon _0}$ is effective. Then $\mathrm {Supp}(D_{\epsilon _0})$ does not contain at least one irreducible component of $\mathrm {Supp}(R)$. Moreover, if (*S*, *D*) is not log canonical at *P*, and (*S*, *R*) is log canonical at *P*, then $(S,D_{\epsilon _0})$ is not log canonical at *P* by Definition 2.1, because

$$\begin{aligned} D=\frac{1}{1+\epsilon _0}D_{\epsilon _0}+\frac{\epsilon _0}{1+\epsilon _0} R \end{aligned}$$

and $\frac{1}{1+\epsilon _0}+\frac{\epsilon _0}{1+\epsilon _0}=1$. Similarly, if the log pair (*S*, *D*) is not Kawamata log terminal at *P*, and (*S*, *R*) is Kawamata log terminal at *P*, then $(S,D_{\epsilon _0})$ is not Kawamata log terminal at *P*.

The following result is well known.

### Lemma 2.5

([4, Exercise 6.18]) If (*S*, *D*) is not log canonical at *P*, then $\mathrm {mult}_{P}(D)>1$. Similarly, if (*S*, *D*) is not Kawamata log terminal at *P*, then $\mathrm {mult}_{P}(D)\geqslant 1$.

Combining with

### Lemma 2.6

([4, Lemma 5.36]) Suppose that *S* is a smooth surface in ${\mathbb {P}}^3$, and $D\sim _{{\mathbb {Q}}} H_{S}$, where $H_S$ is a hyperplane section of *S*. Then each $a_i$ does not exceed 1.

Lemma 2.5 gives

### Corollary 2.7

Suppose that *S* is a smooth surface in ${\mathbb {P}}^3$, and $D\sim _{{\mathbb {Q}}} H_{S}$, where $H_S$ is a hyperplane section of *S*. Then (*S*, *D*) is log canonical outside of finitely many points.

The following result is a special case of a much more general result, which is known as Shokurov’s connectedness principle (see, for example, [4, Theorem 6.3.2]).

### Lemma 2.8

([11, Theorem 6.9]) If $-(K_S+D)$ is big and nef, then the locus where (*S*, *D*) is not Kawamata log terminal is connected.

### Corollary 2.9

Let $C_d$ be a reduced curve in ${\mathbb {P}}^2$ of degree *d*, and let *O* and *Q* be two points in $C_d$ such that $O\ne Q$. If $\mathrm {lct}_{O}({\mathbb {P}}^2,C_d)<\frac{3}{d}$, then $\mathrm {lct}_{Q}({\mathbb {P}}^2,C_d)\geqslant \frac{3}{d}$.

Let $\pi _1:S_1\rightarrow S$ be a blow up of the point *P*, and let $E_1$ be the $\pi _1$-exceptional curve. Denote by $D^1$ the proper transform of the divisor *D* on the surface $S_1$ via $\pi _1$. Then the log pair $(S_1, D^1+(\mathrm {mult}_{P}(D)-1)E_1)$ is often called *the log pull back* of the log pair (*S*, *D*), because

$$\begin{aligned} K_{S_1}+D^1+\Big (\mathrm {mult}_{P}(D)-1\Big )E_1\sim _{{\mathbb {Q}}}\pi _1^{*}\big (K_{S}+D\big ). \end{aligned}$$

This ${\mathbb {Q}}$-rational equivalence implies that the log pair (*S*, *D*) is not log canonical at *P* provided that $\mathrm {mult}_{P}(D)>2$. Similarly, if $\mathrm {mult}_{P}(D)\geqslant 2$, then the singularities of the log pair (*S*, *D*) are not Kawamata log terminal at the point *P*.

### Remark 2.10

The log pair (*S*, *D*) is log canonical at *P* if and only if $(S_1, D^1+(\mathrm {mult}_{P}(D)-1)E_1)$ is log canonical at every point of the curve $E_1$. Similarly, the log pair (*S*, *D*) is Kawamata log terminal at *P* if and only if $(S_1, D^1+(\mathrm {mult}_{P}(D)-1)E_1)$ is Kawamata log terminal at every point of the curve $E_1$.

Let *Z* be an irreducible curve on *S* that contains *P*. Suppose that *Z* is smooth at *P*, and *Z* is not contained in $\mathrm {Supp}(D)$. Let $\mu$ be a non-negative rational number. The following result is a very special case of a much more general result known as *Inversion of Adjunction* (see, for example, [11, § 3.4] or [4, Theorem 6.29]).

### Theorem 2.11

([11, Corollary 3.12], [4, Exercise 6.31], [2, Theorem 7]) Suppose that the log pair $(S,\mu Z+D)$ is not log canonical at *P* and $\mu \leqslant 1$. Then $\mathrm {mult}_{P}(D\cdot Z)>1$.

This result implies

### Theorem 2.12

Suppose that $(S,\mu Z+D)$ is not Kawamata log terminal at *P*, and $\mu <1$. Then $\mathrm {mult}_{P}(D\cdot Z)>1$.

### Proof

The log pair $(S, Z+D)$ is not log canonical at *P*, because $\mu <1$, and $(S,\mu Z+D)$ is not Kawamata log terminal at *P*. Then $\mathrm {mult}_{P}(D\cdot Z)>1$ by Theorem 2.11. $\square$

Theorems 2.11 and 2.12 imply

### Lemma 2.13

If (*S*, *D*) is not log canonical at *P* and $\mathrm {mult}_{P}(D)\leqslant 2$, then there exists a *unique* point in $E_1$ such that $(S_1, D^1+(\mathrm {mult}_{P}(D)-1)E_1)$ is not log canonical at it. Similarly, if (*S*, *D*) is not Kawamata log terminal at *P*, and $\mathrm {mult}_{P}(D)<2$, then there exists a *unique* point in $E_1$ such that $(S_1, D^1+(\mathrm {mult}_{P}(D)-1)E_1)$ is not Kawamata log terminal at it.

### Proof

If $\mathrm {mult}_{P}(D)\leqslant 2$ and $(S_1, D^1+(\lambda \mathrm {mult}_{P}(D)-1)E_1)$ is not log canonical at two distinct points $P_1$ and ${\widetilde{P}}_1$, then

$$\begin{aligned} 2\geqslant \mathrm {mult}_{P}\big (D\big )=D^1\cdot E_1\geqslant \mathrm {mult}_{P_1}\Big (D^1\cdot E_1\Big )+\mathrm {mult}_{{\widetilde{P}}_1}\Big (D^1\cdot E_1\Big )>2 \end{aligned}$$

by Theorem 2.11. By Remark 2.10, this proves the first assertion. Similarly, we can prove the second assertion using Theorem 2.12 instead of Theorem 2.11. $\square$

The following result can be proved similarly to the proof of Lemma 2.5. Let us show how to prove it using Theorem 2.12.

### Lemma 2.14

Suppose that (*S*, *D*) is not Kawamata log terminal at *P*, and (*S*, *D*) is Kawamata log terminal in a punctured neighbourhood of the point *P*, then $\mathrm {mult}_{P}(D)>1$.

### Proof

By Remark 2.10, the log pair $(S_1, D^1+(\mathrm {mult}_{P}(D)-1)E_1)$ is not Kawamata log terminal at some point $P_1\in E_1$. Moreover, if $\mathrm {mult}_{P}(D)<2$, then $(S_1, D^1+(\mathrm {mult}_{P}(D)-1)E_1)$ is Kawamata log terminal at a punctured neighbourhood of the point $P_1$. Thus, if $\mathrm {mult}_{P}(D)\leqslant 1$, then $\mathrm {mult}_{P}\big (D\big )=D^1\cdot E_1>1$ by Theorem 2.12, which is absurd. $\square$

Let $Z_1$ and $Z_2$ be two irreducible curves on the surface *S* such that $Z_1$ and $Z_2$ are not contained in $\mathrm {Supp}(D)$. Suppose that $P\in Z_1\cap Z_2$, the curves $Z_1$ and $Z_2$ are smooth at *P*, the curves $Z_1$ and $Z_2$ intersect each other transversally at *P*. Let $\mu _1$ and $\mu _2$ be non-negative rational numbers.

### Theorem 2.15

([2, Theorem 13]) Suppose that the log pair $(S, \mu _1Z_1+\mu _2Z_2+D)$ is not log canonical at the point *P*, and $\mathrm {mult}_{P}(D)\leqslant 1$. Then either $\mathrm {mult}_{P}(D\cdot Z_{1})>2(1-\mu _{2})$ or $\mathrm {mult}_{P}(D\cdot Z_{2})>2(1-\mu _{1})$ (or both).

This result implies

### Theorem 2.16

Suppose that $(S, \mu _1Z_1+\mu _2Z_2+D)$ is not Kawamata log terminal at *P*, and $\mathrm {mult}_{P}(D)<1$. Then either $\mathrm {mult}_{P}(D\cdot Z_{1})\geqslant 2(1-\mu _{2})$ or $\mathrm {mult}_{P}(D\cdot Z_{2})\geqslant 2(1-\mu _{1})$ (or both).

### Proof

Let $\lambda$ be a rational number such that

$$\begin{aligned} \frac{1}{\mathrm {mult}_{P}(D)}\geqslant \lambda >1. \end{aligned}$$

Then $(S,D+\lambda \mu _1Z_1+\lambda \mu _2Z_2)$ is not log canonical at *P*. Now it follows from Theorem 2.15 that either $\mathrm {mult}_{P}(D\cdot Z_{1})>2(1-\lambda \mu _{2})$ or $\mathrm {mult}_{P}(D\cdot Z_{2})>2(1-\lambda \mu _{1})$ (or both). Since we can choose $\lambda$ to be as close to 1 as we wish, this implies that either $\mathrm {mult}_{P}(D\cdot Z_{1})\geqslant 2(1-\mu _{2})$ or $\mathrm {mult}_{P}(D\cdot Z_{2})\geqslant 2(1-\mu _{1})$ (or both). $\square$

## Reduced Plane Curves

The purpose of this section is to prove Theorems 1.10 and 1.15. Let $C_d$ be a *reduced* plane curve in ${\mathbb {P}}^2$ of degree $d\geqslant 4$, and let *P* be a point in $C_d$. Put $\lambda _1=\frac{2d-3}{d(d-2)}$ and $\lambda _2=\frac{5}{2d}$. To prove Theorem 1.10, we have to show that if the log pair $({\mathbb {P}}^2,\lambda _1 C_d)$ is not Kawamata log terminal at the point *P*, then one of the following assertions hold:

- $$\mathrm {mult}_{P}(C_d)=d$$,
- $C_d$ has singularity ${\mathbb {T}}_{d-1}, {\mathbb {K}}_{d-1}, \widetilde{{\mathbb {T}}}_{d-1}$ or $\widetilde{{\mathbb {K}}}_{d-1}$ at the point *P*,
- $d=4$ and $C_4$ is a Płoski curve (see Definition 1.7).

To prove Theorem 1.15, we have to show that if $({\mathbb {P}}^2,\lambda _2 C_d)$ is not Kawamata log terminal, then either $C_d$ is GIT-unstable or $C_d$ is an even Płoski curve. In the rest of the section, we will do this simultaneously. Let us start with few preliminary results.

### Lemma 3.1

The following inequalities hold:

(i) $$\lambda _1<\frac{2}{d-1}$$,
(ii) $\lambda _1<\frac{2k+1}{kd}$ for every positive integer $k\leqslant d-3$,
(iii) if $d\geqslant 5$, then $\lambda _1<\frac{2k+1}{kd+1}$ for every positive integer $k\leqslant d-4$,
(iv) $$\lambda _1<\frac{3}{d}$$,
(v) $$\lambda _1<\frac{2}{d-2}$$,
(vi) $$\lambda _1<\frac{6}{3d-4}$$,
(vii) if $d\geqslant 5$, then $\lambda _1<\lambda _2$.

### Proof

The equality $\frac{2}{d-1}=\lambda _1+\frac{d-3}{d(d-1)(d-2)}$ implies (i). Let *k* be positive integer. If $k=d-2$, then $\lambda _1=\frac{2k+1}{kd}$. This implies (ii), because $\frac{2k+1}{kd}=\frac{2}{d}+\frac{1}{kd}$ is a decreasing function on *k* for $k\geqslant 1$. Similarly, if $k=d-4$ and $d\geqslant 4$, then $\lambda _1=\frac{2k+1}{kd+1}-\frac{3}{d(d-2)(d^2-4d+1)}<\frac{2k+1}{kd+1}$. This implies (iii), since $\frac{2k+1}{kd+1}=\frac{2}{d}+\frac{d-2}{d(kd+1)}$ is a decreasing function on *k* for $k\geqslant 1$. The equality $\lambda _1=\frac{3}{d}-\frac{d-3}{d(d-2)}$ proves (iv). Note that (v) follows from (i). Since $\frac{6}{3d-4}>\frac{2}{d-1}$, (vi) also follows from (i). Finally, the equality $\lambda _1=\lambda _2-\frac{d-4}{2d(d-2)}$ implies (vii). $\square$

We may assume that $P=[0:0:1]$. Then $C_d$ is given by $F_d(x,y,z)=0$, where $F_{d}(x,y,z)$ is a homogeneous polynomial of degree *d*. Put $x_1=\frac{x}{z}, x_2=\frac{y}{z}$ and $f_d(x_1,x_2)=F_d(x_1,x_2,1)$. Put $m_0 = \mathrm{mult}_P(C_d)$. Then

$$\begin{aligned} f_d\big (x_1,x_2\big )=\sum _{\begin{array}{c} i\geqslant 0, j\geqslant 0,\\ m_0\leqslant i+j\leqslant d \end{array}} \epsilon _{ij}x_1^ix_2^j, \end{aligned}$$

where each $\epsilon _{ij}$ is a complex number. For every positive integers *a* and *b*, define the weight of the polynomial $f_d(x_1,x_2)$ as

$$\begin{aligned} \mathrm {wt}_{(a,b)}\big (f_d(x_1,x_2)\big )=\min \Big \{ai+bj\ \Big \vert \ \epsilon _{ij}\ne 0\Big \}. \end{aligned}$$

Then the Hilbert–Mumford criterion implies

### Lemma 3.2

([7, Lemma 2.1]) Let *a* and *b* be positive integers. If $C_d$ is GIT-stable, then

$$\begin{aligned} \mathrm {wt}_{(a,b)}\Big (f_d\big (x_1,x_2\big )\Big )<\frac{d}{3}\big (a+b\big ). \end{aligned}$$

Similarly, if $C_d$ is GIT-semistable, then $\mathrm {wt}_{(a,b)}(f_d(x_1,x_2))\leqslant \frac{d}{3}(a+b)$.

Let $f_1:S_1\rightarrow {\mathbb {P}}^2$ be a blow up of the point *P*. Denote by $E_1$ the exceptional curve of the blow up $f_1$. Denote by $C^1_d$ the proper transform on $S_1$ of the curve $C_d$.

### Lemma 3.3

If $\mathrm {mult}_{P}(C_d)>\frac{2d}{3}$, then $C_d$ is GIT-unstable. Let *O* be a point in $E_1$. If

$$\begin{aligned} \mathrm {mult}_{P}(C_d)+\mathrm {mult}_{O}(C^1_d)>d, \end{aligned}$$

then $C_d$ is GIT-unstable.

### Proof

Since $\mathrm {mult}_{P}(C_d)=\mathrm {wt}_{(1,1)}(f_d(x_1,x_2))$, the first assertion follows from Lemma 3.2. Let us prove the second assertion. We may assume that *O* is contained in the proper transform of the line in ${\mathbb {P}}^2$ that is given by $x=0$. Then

$$\begin{aligned} \mathrm {wt}_{(2,1)}\big (f_d(x_1,x_2)\big )=\mathrm {mult}_{P}(C_d)+\mathrm {mult}_{O}(C^1_d), \end{aligned}$$

so that the second assertion also follows from Lemma 3.2. $\square$

Now we are ready to prove Theorems 1.10 and 1.15. To do this, we may assume that $C_d$ is not a union of *d* lines passing through the point *P*. Suppose, in addition, that

(**A**): either $({\mathbb {P}}^2,\lambda _1 C_d)$ is not Kawamata log terminal at *P*,
(**B**): or $({\mathbb {P}}^2,\lambda _2 C_d)$ is not Kawamata log terminal at *P*.

We will show that (**A**) implies that either $C_d$ has singularity ${\mathbb {T}}_{d-1}, {\mathbb {K}}_{d-1}, \widetilde{{\mathbb {T}}}_{d-1}$ or $\widetilde{{\mathbb {K}}}_{d-1}$ at the point *P*, or $C_d$ is a Płoski quartic curve. Similarly, we will show that (**B**) implies that either $C_d$ is GIT-unstable (i.e. $C_d$ is not GIT-semistable), or $C_d$ is an even Płoski curve. If (**A**) holds, let $\lambda =\lambda _1$. If (**B**) holds, let $\lambda =\lambda _2$.

If $d=4$, then $\lambda _1=\lambda _2$. If $d\geqslant 5$, then $\lambda _1<\lambda _2$ by Lemma 3.1(vii). Since $C_d$ is reduced and $\lambda <1$, the log pair $({\mathbb {P}}^2,\lambda C_d)$ is Kawamata log terminal outside of finitely many points. Thus, it is Kawamata log terminal outside of *P* by Lemma 2.8.

Then the log pair $(S_1,\lambda C^1_d+(\lambda m_0-1)E_1)$ is not Kawamata log terminal at some point $P_1\in E_1$ by Remark 2.10. Note that we have

$$\begin{aligned} K_{S_1}+\lambda C^1_d+\Big (\lambda m_0-1\Big )E_1\sim _{{\mathbb {Q}}}f_1^*\Big (K_{{\mathbb {P}}^2}+\lambda C_d\Big ). \end{aligned}$$

Let $f_2:S_2\rightarrow S_1$ be a blow up of the point $P_1$, and let $E_2$ be its exceptional curve. Denote by $C^2_d$ the proper transform on $S_2$ of the curve $C_d$, and denote by $E_1^2$ the proper transform on $S_2$ of the curve $E_1$. Put $m_1=\mathrm {mult}_{P_1}(C_d^1)$. Then

$$\begin{aligned}&K_{S_2}+\lambda C^2_d+\big (\lambda m_0-1\big )E_1^2+\big (\lambda (m_0+m_1)-2\big )E_2\sim _{{\mathbb {Q}}} f_2^*\Big (K_{S_1} +\lambda C^1_d\\&\quad +\,\big (\lambda m_0-1\big )E_1\Big ). \end{aligned}$$

By Remark 2.10, the log pair $(S_2,\lambda C^2_d+(\lambda m_0-1)E_1^2+(\lambda (m_0+m_1)-2)E_2)$ is not Kawamata log terminal at some point $P_2\in E_2$. Let $f_3:S_3\rightarrow S_2$ be a blow up of this point, and let $E_3$ be the $f_3$-exceptional curve. Denote by $C^3_d$ the proper transform on $S_3$ of the curve $C_d$, denote by $E_1^3$ the proper transform on $S_3$ of the curve $E_1$, and denote by $E_2^3$ the proper transform on $S_3$ of the curve $E_2$. Put $m_2=\mathrm {mult}_{P_2}(C_d^2)$. Then

$$\begin{aligned}&K_{S_3}+\lambda _2 C^3_d+\big (\lambda _2 m_0-1\big )E_1^3\\&\quad +\,\big (\lambda _2(m_0+m_1)-2\big )E_2^3+\big (\lambda _2(2m_0+m_1+m_2)-4\big )E_3\sim _{{\mathbb {Q}}}\\&\quad \sim _{{\mathbb {Q}}} f_3^*\Big (K_{S_2}+\lambda _2 C^2_d+\big (\lambda _2 m_0-1\big )E_1^2+\big (\lambda _2(m_0+m_1)-2\big )E_2\Big ). \end{aligned}$$

Thus, the log pair $(S_3,\lambda _2 C^3_d+(\lambda _2 m_0-1)E_1^3+(\lambda _2(m_0+m_1)-2)E_2^3+(\lambda _2(2m_0+m_1+m_2)-4)E_3)$ is not Kawamata log terminal at some point $P_3\in E_3$ by Remark 2.10. Note that the divisor $\lambda _2 C^3_d+(\lambda _2 m_0-1)E_1^3+(\lambda _2(m_0+m_1)-2)E_2^3+(\lambda _2(2m_0+m_1+m_2)-4)E_3$ is effective by Lemma 2.5.

### Lemma 3.4

One has $$\lambda m_0<2$$.

### Proof

Since $C_d$ is not a union of *d* lines passing through *P*, we have $m_0\leqslant d-1$. Thus, if (**A**) holds, then $\lambda m_0<2$ by Lemma 3.1(i), because $d\geqslant 4$. Similarly, if (**B**) holds, then $m_0\leqslant \frac{2d}{3}$ by Lemma 3.3, which implies that $\lambda m_0\leqslant \frac{10}{6}<2$. $\square$

Thus, the log pair $(S_1,\lambda C^1_d+(\lambda m_0-1)E_1)$ is Kawamata log terminal outside of $P_1$ by Lemma 2.13. Note that $P_1\in C^1_d$, because the log pair $(S_1, (\lambda m_0-1)E_1)$ is Kawamata log terminal at $P_1$. Thus, we have $m_1>0$.

Let *L* be the line in ${\mathbb {P}}^2$ whose proper transform on $S_1$ contains the point $P_1$. Such a line exists and it is unique. By a suitable linear change of coordinates, we may assume that *L* is given by $x=0$. Denote by $L^1$ the proper transform of the line *L* on the surface $S_1$.

### Lemma 3.5

Suppose that (**A**) holds and $m_0=d-1$. Then $C_d$ has singularity ${\mathbb {K}}_{d-1}, \widetilde{{\mathbb {K}}}_{d-1}, {\mathbb {T}}_{d-1}$ or $\widetilde{{\mathbb {T}}}_{d-1}$ at the point *P*.

### Proof

Suppose that *L* is not an irreducible component of the curve $C_d$. Then $m_0+m_1\leqslant d$, because

$$\begin{aligned} d-1-m_0=C_d^1\cdot L^1\geqslant m_1. \end{aligned}$$

Since $m_0=d-1$, this gives $m_1=1$. Then $P_1\in C_{d}^1$ and the curve $C_{d}^1$ is smooth at $P_1$. Put $k=\mathrm {mult}_{P_1}(C_d^1\cdot E_1)$. Applying Corollary 2.3 to the log pair $(S_1,\lambda _1 C^1_d+(\lambda _1 m_0-1)E_1)$ at the point $P_1$, we get

$$\begin{aligned} k\lambda _1 m_0\geqslant k+1, \end{aligned}$$

which gives $\lambda _1\geqslant \frac{2k+1}{kd}$. Then $k\geqslant d-2$ by Lemma 3.1(ii). Since

$$\begin{aligned} k\leqslant C_d^1\cdot E_1=m_0=d-1, \end{aligned}$$

either $k=d-1$ or $k=d-2$. If $k=d-1$, then $C_d$ has singularity ${\mathbb {K}}_{d-1}$ at *P*. If $k=d-2$, then $C_d$ has singularity $\widetilde{{\mathbb {K}}}_{d-1}$ at the point *P*.

To complete the proof, we may assume that *L* is an irreducible component of the curve $C_d$. Then $C_{d}=L+C_{d-1}$, where $C_{d-1}$ is a reduced curve in ${\mathbb {P}}^2$ of degree $d-1$ such that *L* is not its irreducible component. Denote by $C_{d-1}^1$ its proper transform on $S_1$. Put $n_0=\mathrm {mult}_{P}(C_{d-1})$ and $n_1=\mathrm {mult}_{P_1}(C_{d-1}^1)$. Then $n_0=m_0-1=d-2$ and $n_1=m_1-1$. This implies that $P_1\in C_{d-1}^1$, since the log pair $(S_1,\lambda _1 L^1+(\lambda _1 m_0-1)E_1)$ is Kawamata log terminal at *P*. Hence, $n_1\geqslant 1$. One the other hand, we have

$$\begin{aligned} d-1-n_0=C_{d-1}^1\cdot L^1\geqslant n_1, \end{aligned}$$

which implies that $n_0+n_1\leqslant d-1$. Then $n_1=1$, since $n_0=d-2$.

We have $P_1\in C_{d-1}^1$ and $C_{d-1}^1$ is smooth at $P_1$. Moreover, since

$$\begin{aligned} 1=d-1-n_0=L^1\cdot C_{d-1}^1\geqslant n_1=1, \end{aligned}$$

the curve $C_{d-1}^1$ intersects the curve $L^1$ transversally at the point $P_1$. Put $k=\mathrm {mult}_{P_1}(C_{d-1}^1\cdot E_1)$. Then $k\geqslant 1$. Applying Lemma 2.2 to the log pair $(S_1,\lambda _1 C^1_{d-1}+\lambda _1 L^1+(\lambda _1 (n_0+1)-1)E_1)$ at the point $P_1$, we get

$$\begin{aligned} k\Big (\lambda _1(n_0+2)-1\Big )+\lambda _1\geqslant k+1. \end{aligned}$$

Then $\lambda _1\geqslant \frac{2k+1}{kd+1}$. Then $k\geqslant d-3$ by Lemma 3.1(iii). Since

$$\begin{aligned} k\leqslant E_1\cdot C_{d-1}^1=n_0=d-2, \end{aligned}$$

either $k=d-2$ or $k=d-3$. In the former case, $C_d$ has singularity ${\mathbb {T}}_{d-1}$ at the point *P*. In the latter case, $C_d$ has singularity $\widetilde{{\mathbb {T}}}_{d-1}$ at the point *P*. $\square$

### Lemma 3.6

Suppose that (**A**) holds and $m_0\leqslant d-2$. Then the line *L* is not an irreducible component of the curve $C_d$.

### Proof

Suppose that *L* is an irreducible component of the curve $C_d$. Let us see for a contradiction. Put $C_{d}=L+C_{d-1}$, where $C_{d-1}$ is a reduced curve in ${\mathbb {P}}^2$ of degree $d-1$ such that *L* is not its irreducible component. Denote by $C_{d-1}^1$ its proper transform on $S_1$. Put $n_0=\mathrm {mult}_{P}(C_{d-1})$ and $n_1=\mathrm {mult}_{P_1}(C_{d-1}^1)$. Then $(S_1, (\lambda _1(n_0+1)-1)E_1+\lambda _1 L^1+\lambda _1 C_{d-1}^1)$ is not Kawamata log terminal at $P_1$ and is Kawamata log terminal outside of the point $P_1$. In particular, $n_1\ne 0$, because $(S_1, (\lambda _1(n_0+1)-1)E_1+\lambda _1 L^1)$ is Kawamata log terminal at $P_1$. On the other hand,

$$\begin{aligned} d-1-n_0=L^1\cdot C_{d-1}^1\geqslant n_1, \end{aligned}$$

which implies that $n_0+n_1\leqslant d-1$. Furthermore, we have $n_0=m_0-1\leqslant d-3$.

Since $n_0+n_1\geqslant 2n_1$, we have $n_1\leqslant \frac{d-1}{2}$. Then $\lambda n_1<1$ by Lemma 3.1(i). Thus, we can apply Theorem 2.16 to the log pair $(S_1, (\lambda _1(n_0+1)-1)E_1+\lambda _1 L^1+\lambda _1 C_{d-1}^1)$ at the point $P_1$. This gives either

$$\begin{aligned} \lambda _1\big (d-1-n_0\big )=\lambda _1 C_{d-1}^1\cdot L^1\geqslant 2\Big (2-\lambda _1\big (n_0+1\big )\Big ) \end{aligned}$$

or

$$\begin{aligned} \lambda _1 n_0=\lambda _1 C_{d-1}^1\cdot E_1\geqslant 2\big (1-\lambda _1\big ) \end{aligned}$$

(or both). In the former case, we have $\lambda _1(d+1+n_0)\geqslant 4$. In the latter case, we have $\lambda _1(n_0+2)>2$. Thus, in both cases we have $\lambda _1(d-1)\geqslant 2$, since $n_0\leqslant d-3$. But $\lambda _1(d-1)<2$ by Lemma 3.1(i). This is a contradiction. $\square$

If the curve $C_d$ is GIT-semistable, then $m_0\leqslant d-2$ by Lemma 3.3. Thus, it follows from Lemma 3.5 that we may assume that

$$\begin{aligned} m_0\leqslant d-2 \end{aligned}$$

in order to complete the proof of Theorems 1.10 and 1.15. Moreover, if *L* is not an irreducible component of the curve $C_d$, then

$$\begin{aligned} d-m_0=C_d^1\cdot L^1\geqslant m_1. \end{aligned}$$

Thus, if (**A**) holds, then $m_0+m_1\leqslant d$ by Lemma 3.6. Similarly, if the curve $C_d$ is GIT-semistable, then $m_0+m_1\leqslant d$ by Lemma 3.3. Thus, to complete the proof of Theorems 1.10 and 1.15, we may also assume that

3.1 $$\begin{aligned} m_0+m_1\leqslant d. \end{aligned}$$

Then $\lambda (m_0+m_1)<3$ by Lemma 3.1(v), so that $(S_2,\lambda C^2_d+(\lambda m_0-1)E_1^2+(\lambda (m_0+m_1)-2)E_2)$ is Kawamata log terminal outside of the point $P_2$ by Lemma 2.13. Furthermore, we have

### Lemma 3.7

Suppose that $P_2=E_1^2\cap E_2$. Then (**A**) does not hold and $C_d$ is GIT-unstable.

### Proof

We have $m_0-m_1=E_1^2\cdot C_d^2\geqslant m_2$, so that

3.2 $$\begin{aligned} m_2\leqslant \frac{m_0}{2}, \end{aligned}$$

because $2m_2\leqslant m_1+m_2$. On the other hand, $m_0\leqslant d-2$ by assumption. Thus, we have $m_2\leqslant \frac{d-2}{2}$.

Suppose that (**A**) holds. Then $\lambda =\lambda _1$ and $\lambda _1 m_2<1$ by Lemma 3.1(v). Thus, we can apply Theorem 2.16 to the log pair $(S_2,\lambda _1 C^2_d+(\lambda _1 m_0-1)E_1^2+(\lambda _1 (m_0+m_1)-2)E_2)$. This gives either

$$\begin{aligned} \lambda _1\big (m_0-m_1\big )=\lambda _1 C_{d}^2\cdot E_1^2\geqslant 2\Big (3-\lambda _1\big (m_0+m_1\big )\Big ) \end{aligned}$$

or

$$\begin{aligned} \lambda _1 m_1=\lambda _1 C_{d}^2\cdot E_2\geqslant 2\big (2-\lambda _1 m_0\big ) \end{aligned}$$

(or both). The former inequality implies $\lambda _1(3m_0+m_1)\geqslant 6$. The latter inequality implies $\lambda _1(2m_0+m_1)\geqslant 4$. On the other hand, $m_0+m_1\leqslant d$ by (3.1), and $m_0\leqslant d-2$ by assumption. Thus, $3m_0+m_1\leqslant 3d-4$ and $2m_0+m_1\leqslant 2d-2$. Then $\lambda _1(3m_0+m_1)<6$ by Lemma 3.1(vi), and $\lambda _1(2m_0+m_1)<4$ by Lemma 3.1(i). The obtained contradiction shows that (**A**) does not hold.

We see that (**B**) holds. We have to show that $C_d$ is GIT-unstable. Suppose that this is not the case, so that $C_d$ is GIT-semistable. Let us seek for a contradiction.

By Lemma 3.2, we have $2m_0+m_1+m_2\leqslant \frac{5d}{3}$, because

$$\begin{aligned} \mathrm {wt}_{(3,2)}\Big (f_d\big (x_1,x_2\big )\Big )=2m_0+m_1+m_2. \end{aligned}$$

Thus, we have $\lambda _2(2m_0+m_1+m_2)-4<1$ by Lemma 3.1(v). Hence, the log pair $(S_3,\lambda _2 C^3_d+(\lambda _2 m_0-1)E_1^3+(\lambda _2(m_0+m_1)-2)E_2^3+(\lambda _2(2m_0+m_1+m_2)-4)E_3)$ is Kawamata log terminal outside of the point $P_3$ by Remark 2.10.

If $P_3=E_1^3\cap E_3$, then it follows from Theorem 2.12 that

$$\begin{aligned} \lambda _2\big (m_0-m_1-m_2\big )=\lambda _2 C_d^3\cdot E_1^3>5-\lambda _2\big (2m_0+m_1+m_2\big ), \end{aligned}$$

which implies that $m_0>\frac{5}{3\lambda _2}=\frac{2d}{3}$, which is impossible by Lemma 3.3. If $P_3=E_2^3\cap E_3$, then it follows from Theorem 2.12 that

$$\begin{aligned} \lambda _2\big (m_1-m_2\big )=\lambda _2C_d^3\cdot E_2^3>5-\lambda _2\big (2m_0+m_1+m_2\big ), \end{aligned}$$

which implies that $m_0+m_1>\frac{5}{2\lambda _2}=d$, which is impossible by Lemma 3.3. Thus, we see that $P_3\not \in E_1^3\cup E_2^3$. Then the log pair $(S_3,\lambda _2 C^3_d+(\lambda _2(2m_0+m_1+m_2)-4)E_3)$ is not Kawamata log terminal at $P_3$. Hence, Theorem 2.12 gives

$$\begin{aligned} \lambda _2 m_2=\lambda _2 C_d^3\cdot E_3>1, \end{aligned}$$

which implies that $m_2>\frac{1}{\lambda _2}=\frac{2d}{5}$. Then $m_0>\frac{4d}{5}$ by (3.2), which is impossible by Lemma 3.3. $\square$

Thus, to complete the proof of Theorems 1.10 and 1.15, we may assume that

$$\begin{aligned} P_2\ne E_1^2\cap E_2. \end{aligned}$$

Denote by $L^2$ the proper transform of the line *L* on the surface $S_2$.

### Lemma 3.8

One has $$P_2\ne L^2\cap E_2$$.

### Proof

Suppose that $P_2=L^2\cap E_2$. If *L* is not an irreducible component of the curve $C_d$, then

$$\begin{aligned} d-m_0-m_1=L^2\cdot E_2\geqslant m_2, \end{aligned}$$

which implies that $m_0+m_1+m_2\leqslant d$. Thus, if (**A**) holds, then $\lambda =\lambda _1$ and *L* is not an irreducible component of the curve $C_d$ by Lemma 3.6, which implies that

$$\begin{aligned} \lambda _1 d\geqslant \lambda _1\big (m_0+m_1+m_2\big )>3 \end{aligned}$$

by Lemma 2.14. On the other hand, $\lambda _1 d<3$ by Lemma 3.1(iv). This shows that (**B**) holds.

Since $\lambda =\lambda _2=\frac{5}{2d}<\frac{3}{d}$ and $\lambda _2(m_0+m_1+m_2)>3$ by Lemma 2.14, we have $m_0+m_1+m_2>d$. In particular, the line *L* must be an irreducible component of the curve $C_d$.

Put $C_{d}=L+C_{d-1}$, where $C_{d-1}$ is a reduced curve in ${\mathbb {P}}^2$ of degree $d-1$ such that *L* is not its irreducible component. Denote by $C_{d-1}^1$ its proper transform on $S_1$, and denote by $C_{d-1}^2$ its proper transform on $S_2$. Put $n_0=\mathrm {mult}_{P}(C_{d-1}), n_1=\mathrm {mult}_{P_1}(C_{d-1}^1)$ and $n_2=\mathrm {mult}_{P_2}(C_{d-1}^2)$. Then $(S_2, (\lambda _2(n_0+n_1+2)-2)E_2+\lambda _2 L^1+\lambda _2 C_{d-1}^1)$ is not Kawamata log terminal at $P_2$ and is Kawamata log terminal outside of the point $P_2$. Then Theorem 2.12 implies

$$\begin{aligned} \lambda _2\big (d-1-n_0-n_1\big )= & {} \lambda _2 C_{d-1}^2\cdot L^2>1-\big (\lambda _2(n_0+n_1+2)-2\big )\\= & {} 3-\lambda _2(n_0+n_1+2), \end{aligned}$$

which implies that $\frac{5(d+1)}{2d}=\lambda _2(d+1)>3$. Hence, $d=4$. Then $\lambda =\lambda _2=\frac{5}{8}$.

By (3.1), $n_0+n_1\leqslant 2$. Thus, $n_0=n_1=n_2=1$, since

$$\begin{aligned} \frac{5}{8}\big (n_0+n_1+n_2+3\big )=\lambda _2\big (m_0+m_1+m_2\big )>3 \end{aligned}$$

by Lemma 2.14. Then $C_{3}$ is a irreducible cubic curve that is smooth at *P*, the line *L* is tangent to the curve $C_3$ at the point *P*, and *P* is an inflexion point of the cubic curve $C_3$. This implies that $\mathrm {lct}_P({\mathbb {P}}^2, C_d)=\frac{2}{3}$. Since $\frac{2}{3}>\frac{5}{8}=\lambda _2$, the log pair $({\mathbb {P}}^2, \lambda _2 C_d)$ must be Kawamata log terminal at the point *P*, which contradicts (**B**). $\square$

Recall that $m_0+m_1\leqslant d$ by (3.1). Then $m_1\leqslant \frac{d}{2}$, since $2m_1\leqslant m_0+m_1$. Thus, we have

3.3 $$\begin{aligned} \lambda \big (m_0+m_1+m_2\big )\leqslant \lambda \big (m_0+2m_1\big )\leqslant \lambda \frac{3d}{2}\leqslant \lambda _2\frac{3d}{2}=\frac{15}{4}<4. \end{aligned}$$

Therefore, the log pair $(S_3,\lambda C^3_d+(\lambda (m_0+m_1)-2)E_2^3+(\lambda (m_0+m_1+m_2)-3)E_3)$ is Kawamata log terminal outside of the point $P_3$ by Lemma 2.13.

### Lemma 3.9

One has $$P_3\ne E_2^3\cap E_3$$.

### Proof

If $P_3=E_2^3\cap E_3$, then Theorem 2.12 gives

$$\begin{aligned} \lambda \big (m_1-m_2\big )=\lambda C_d^3\cdot E_2^3>1-\Big (\lambda \big (m_0+m_1+m_2\big )-3\Big )=4-\lambda \big (m_0+m_1+m_2\big ), \end{aligned}$$

which implies that $\lambda (m_0+2m_1)>4$. But $\lambda (m_0+2m_1)<4$ by (3.3). $\square$

Let $f_4:S_4\rightarrow S_3$ be a blow up of the point $P_3$, and let $E_4$ be its exceptional curve. Denote by $C^4_d$ the proper transform on $S_4$ of the curve $C_d$, denote by $E_3^4$ the proper transform on $S_4$ of the curve $E_3$, and denote by $L^4$ the proper transform of the line *L* on the surface $S_4$. Then $(S_4,\lambda C^4_d+(\lambda (m_0+m_1+m_2)-3)E_3^4+(\lambda (m_0+m_1+m_2+m_3)-4)E_4)$ is not Kawamata log terminal at some point $P_4\in E_4$ by Remark 2.10. Moreover, we have

$$\begin{aligned}&2L^4+E_1^4+2E_2^4+E_3^4\sim (f_1\circ f_2\circ f_3\circ f_4)^*\Big ({\mathcal {O}}_{{\mathbb {P}}^2}\big (2\big )\Big )-(f_2\circ f_3\circ f_4)^*\big (E_1\big )\\&\quad -(f_3\circ f_4)^*\big (E_2\big )-f_4^*\big (E_3\big )-E_4. \end{aligned}$$

### Lemma 3.10

The linear system $|2L^4+E_1^4+2E_2^4+E_3^4|$ is a pencil that does not have base points. Moreover, every divisor in $|2L^4+E_1^4+2E_2^4+E_3^4|$ that is different from $2L^4+E_1^4+2E_2^4+E_3^4$ is a smooth curve whose image on ${\mathbb {P}}^2$ is a smooth conic that is tangent to *L* at the point *P*.

### Proof

All assertions follows from $P_2\not \in E_1^2\cup L^2$ and $P_3\not \in E_2^3$. $\square$

Let $C_2^4$ be a general curve in $|2L^4+E_1^4+2E_2^4+E_3^4|$. Denote by $C_2$ its image on ${\mathbb {P}}^2$, and denote by ${\mathcal {L}}$ the pencil generated by 2*L* and $C_2$. Then *P* is the only base point of the pencil ${\mathcal {L}}$, and every conic in ${\mathcal {L}}$ except 2*L* and $C_2$ intersects $C_2$ at *P* with multiplicity 4 (cf. [3, Remark 1.14]).

### Lemma 3.11

One has $m_0+m_1+m_2+m_3\leqslant m_0+m_1+2m_2\leqslant \frac{5}{\lambda }$. If $m_0+m_1+m_2+m_3=\frac{5}{\lambda }$, then *d* is even and $C_d$ is a union of $\frac{d}{2}\geqslant 2$ smooth conics in ${\mathcal {L}}$, where $d=4$ if (**A**) holds.

### Proof

By (3.1), we have $m_2+m_3\leqslant 2m_2\leqslant m_0+m_1\leqslant d$. This gives

$$\begin{aligned} m_0+m_1+m_2+m_3\leqslant m_0+m_1+2m_2\leqslant 2d=\frac{5}{\lambda _2}\leqslant \frac{5}{\lambda }. \end{aligned}$$

To complete the proof, we may assume that $m_0+m_1+m_2+m_3=\frac{5}{\lambda }$. Then all inequalities above must be equalities. Thus, we have $m_2=m_3=\frac{d}{2}$ and $\lambda _1=\lambda _2$. In particular, if (**A**) holds, then $d=4$, because $\lambda _1<\lambda _2=\frac{5}{2d}$ for $d\geqslant 5$ by Lemma 3.1(vii). Moreover, since $m_0\geqslant m_1\geqslant m_2=\frac{d}{2}$ and $m_0+m_1\leqslant d$, we see that $m_0=m_1=\frac{d}{2}$. Thus, *d* is even and

$$\begin{aligned} C_d^4\sim \frac{d}{2}\Big (2L^4+E_1^4+2E_2^4+E_3^4\Big ), \end{aligned}$$

where $d=4$ if (**A**) holds. Since $|2L^4+E_1^4+2E_2^4+E_3^4|$ is a free pencil and $C_d^4$ is reduced, it follows from Lemma 3.10 that $C_d^4$ is a union of $\frac{d}{2}$ smooth curves in $|2L^4+E_1^4+2E_2^4+E_3^4|$. In particular, $L^4$ is not an irreducible component of $C_d^4$. Thus, the curve $C_d$ is a union of $\frac{d}{2}$ smooth conics in ${\mathcal {L}}$, where $d=4$ if (**A**) holds. $\square$

We see that $m_0+m_1+m_2+m_3\leqslant \frac{5}{\lambda }$. Moreover, if $m_0+m_1+m_2+m_3=\frac{5}{\lambda }$, then $C_d$ is an even Płoski curve. Furthermore, if $m_0+m_1+m_2+m_3=\frac{5}{\lambda }$ and (**A**) holds, then $d=4$. Thus, to prove Theorems 1.10 and 1.15, we may assume that

$$\begin{aligned} m_0+m_1+m_2+m_3<\frac{5}{\lambda }. \end{aligned}$$

Let us show that this assumption leads to a contradiction. By Lemma 2.13, this inequality implies that the log pair $(S_4,\lambda C^4_d+(\lambda (m_0+m_1+m_2)-3)E_3^4+(\lambda (m_0+m_1+m_2+m_3)-4)E_4)$ is Kawamata log terminal outside of the point $P_4$.

### Lemma 3.12

One has $$P_4\ne E_3^4\cap E_4$$.

### Proof

By Lemma 3.11, $m_0+m_1+2m_2\leqslant \frac{5}{\lambda }$. If $P_4=E_3^4\cap E_4$, then Theorem 2.12 gives

$$\begin{aligned} \lambda \big (m_2-m_3\big )=\lambda C_d^4\cdot E_3^4>5-\lambda \big (m_0+m_1+m_2+m_3\big ), \end{aligned}$$

which implies that $m_0+m_1+2m_2>\frac{5}{\lambda }$. This shows that $P_4\ne E_3^4\cap E_4$. $\square$

Thus, the log pair $(S_4,\lambda C^4_d+(\lambda (m_0+m_1+m_2+m_3)-4)E_4)$ is not Kawamata log terminal at $P_4$ and is Kawamata log terminal outside of the point $P_4$.

Let $Z^4$ be the curve in $|2L^4+E_1^4+2E_2^4+E_3^4|$ that passes through the point $P_4$. Then $Z^4$ is a smooth irreducible curve by Lemma 3.8. Denote by *Z* the proper transform of this curve on ${\mathbb {P}}^2$. Then *Z* is a smooth conic in the pencil ${\mathcal {L}}$ by Lemma 3.10. If *Z* is not an irreducible component of the curve $C_d$, then

$$\begin{aligned} 2d-\big (m_0+m_1+m_2+m_3\big )=Z^4\cdot C_d^4\geqslant \mathrm {mult}_{P_4}(C_d^4). \end{aligned}$$

On the other hand, it follows from Lemma 2.14 that

$$\begin{aligned} \mathrm {mult}_{P_4}(C_d^4)+m_0+m_1+m_2+m_3>\frac{5}{\lambda }. \end{aligned}$$

This shows that *Z* is an irreducible component of the curve $C_d$, since $\lambda \leqslant \lambda _2=\frac{5}{2d}$.

Put $C_{d}=Z+C_{d-2}$, where $C_{d-2}$ is a reduced curve in ${\mathbb {P}}^2$ of degree $d-2$ such that *Z* is not its irreducible component. Denote by $C_{d-2}^1, C_{d-2}^2, C_{d-2}^3$ and $C_{d-2}^4$ its proper transforms on the surfaces $S_1, S_2, S_3$ and $S_4$, respectively. Put $n_0=\mathrm {mult}_{P}(C_{d-2}), n_1=\mathrm {mult}_{P_1}(C_{d-2}^1), n_2=\mathrm {mult}_{P_2}(C_{d-2}^2), n_3=\mathrm {mult}_{P_3}(C_{d-2}^3)$ and $n_4=\mathrm {mult}_{P_4}(C_{d-2}^4)$. Then

$$\begin{aligned} \Big (S_4, \lambda C_{d-2}^4+\lambda Z^4+(\lambda (n_0+n_1+n_2+n_3+4)-4)E_4\Big ) \end{aligned}$$

is not Kawamata log terminal at $P_4$ and is Kawamata log terminal outside of the point $P_4$. Thus, applying Theorem 2.12, we get

$$\begin{aligned} \lambda \Big (2\big (d-2\big )-n_0-n_1-n_2-n_3\Big )=\lambda C_{d-2}^4\cdot Z^4>5-\lambda \big (n_0+n_1+n_2+n_3+4\big ), \end{aligned}$$

which implies that $\lambda >\frac{5}{2d}$. This is impossible, since $\lambda \leqslant \lambda _2=\frac{5}{2d}$.

The obtained contradiction completes the proof of Theorems 1.10 and 1.15.

## Smooth Surfaces in ${\mathbb {P}}^3$

The purpose of this section is to prove Theorem 1.17. Let *S* be a smooth surface in ${\mathbb {P}}^3$ of degree $d\geqslant 3$, let $H_{S}$ be its hyperplane section, let *P* be a point in *S*, and let $T_P$ be the hyperplane section of the surface *S* that is singular at *P*. Note that $T_P$ is reduced by Lemma 2.6. Put $\lambda =\frac{2d-3}{d(d-2)}$. Then Theorem 1.17 follows from Theorem 1.10, Remark 2.4 and

### Proposition 4.1

Let *D* be any effective ${\mathbb {Q}}$-divisor on *S* such that $D\sim _{{\mathbb {Q}}} H_S$. Suppose that $\mathrm {Supp}(D)$ does not contain at least one irreducible component of the curve $T_P$. Then $(S,\lambda D)$ is log canonical at *P*.

For $d=3$, this result is just [3, Corollary 1.13]. In the remaining part of the section, we will prove Proposition 4.1. Note that we will do this *without* using [3, Corollary 1.13]. Let us start with

### Lemma 4.2

The following assertions hold:

(i) $$\lambda \leqslant \frac{2}{d-1}$$,
(ii) if $d\geqslant 5$, then $\lambda \leqslant \frac{3}{d+1}$,
(iii) if $d\geqslant 5$, then $\lambda \leqslant \frac{4}{d+3}$,
(iv) If $d\geqslant 6$, then $\lambda \leqslant \frac{3}{d+2}$,
(v) $$\lambda \leqslant \frac{4}{d+1}$$,
(vi) $$\lambda \leqslant \frac{3}{d}$$.

### Proof

The equality $\frac{2}{d-1}=\lambda +\frac{d-3}{d(d-1)(d-2)}$ implies (i), $\frac{4}{d+1}=\lambda +\frac{d^2-5d+3}{d(d+1)(d-2)}$ implies (ii), and $\frac{4}{d+3}=\lambda +\frac{2d^2-11d+9}{d(d+3)(d-2)}$ implies (iii). Similarly, (iv) follows from $\frac{3}{d+2}=\lambda +\frac{d^2-7d+6}{d(d^2-4)}$, (v) follows from $\frac{4}{d+1}=\lambda +\frac{2d^2-7d+3}{d(d+1)(d-2)}$, and (vi) follows from $\frac{3}{d}=\lambda +\frac{d-3}{d(d-2)}$. $\square$

Let *n* be the number of irreducible components of the curve $T_P$. Write

$$\begin{aligned} T_P=T_1+\cdots +T_n, \end{aligned}$$

where each $T_i$ is an irreducible curve on the surface *S*. For every curve $T_i$, we denote its degree by $d_i$, and we put $t_i=\mathrm {mult}_{P}(T_i)$.

### Lemma 4.3

Suppose that $n\geqslant 2$. Then

$$\begin{aligned} T_i\cdot T_i=-d_i(d-d_i-1) \end{aligned}$$

for every $T_i$, and $T_i\cdot T_j=d_id_j$ for every $T_i$ and $T_j$ such that $T_i\ne T_j$.

### Proof

The curve $T_P$ is cut out on *S* by a hyperplane $H\subset {\mathbb {P}}^3$. Then $H\cong {\mathbb {P}}^2$. Hence, for every $T_i$ and $T_j$ such that $T_i\ne T_j$, we have $(T_i\cdot T_j)_S=(T_i\cdot T_j)_H=d_id_j$. In particular, we have

$$\begin{aligned} d_1=T_P\cdot T_1=T_1^2+\sum _{i=2}^nT_i\cdot T_1=T_1^2+\sum _{i=2}^n d_id_1=T_1^2+(d-d_1)d_1, \end{aligned}$$

which gives $T_1\cdot T_1=-d_1(d-d_1-1)$. Similarly, we see that $T_i\cdot T_i=-d_i(d-d_i-1)$ for every curve $T_i$. $\square$

Let *D* be any effective ${\mathbb {Q}}$-divisor on *S* such that $D\sim _{{\mathbb {Q}}} H_S$. Write

$$\begin{aligned} D=\sum _{i=1}^{n}a_iT_i+\Delta , \end{aligned}$$

where each $a_i$ is a non-negative rational number, and $\Delta$ is an effective ${\mathbb {Q}}$-divisor on *S* whose support does not contain the curves $T_1,\ldots ,T_n$. To prove Proposition 4.1, it is enough to show that the log pair $(S,\lambda D)$ is log canonical at *P* provided that at least one number among $a_1,\ldots ,a_n$ vanishes.

Without loss of generality, we may assume that $a_n=0$. Suppose that the log pair $(S,\lambda D)$ is not log canonical at *P*. Let us seek for a contradiction.

### Lemma 4.4

Suppose that $n\geqslant 2$. Then

$$\begin{aligned} \sum _{i=1}^{k}a_id_id_n\leqslant d_n-t_n\mathrm {mult}_{P}(\Delta ). \end{aligned}$$

In particular, $\sum _{i=1}^{k}a_id_i\leqslant 1$ and each $a_i$ does not exceed $\frac{1}{d_i}$.

### Proof

One has

$$\begin{aligned} d_n= & {} T_n\cdot D=T_n\cdot \Bigg (\sum _{i=1}^{n}a_iT_i+\Delta \Bigg )=\sum _{i=1}^{n}a_id_id_n+T_n\cdot \Delta \\\geqslant & {} \sum _{i=1}^{n}a_id_id_n+t_n\mathrm {mult}_{P}(\Delta ), \end{aligned}$$

which implies the required inequality. $\square$

Put $$m_0=\mathrm {mult}_{P}(D)$$.

### Lemma 4.5

Suppose that $P\in T_n$. Then $d_n>\frac{d-1}{2}$. If $n\geqslant 2$, then $T_n$ is smooth at *P*.

### Proof

Since $T_n$ is not contained in the support of the divisor *D*, we have

$$\begin{aligned} d\geqslant d_n=T_n\cdot D\geqslant t_nm_0, \end{aligned}$$

which implies that $m_0\leqslant \frac{d_n}{t_n}$. Since $m_0>\frac{1}{\lambda }$ by Lemma 2.5, we have $d_n>\frac{d-1}{2}$ by Lemma 4.2(i). Moreover, if $n\geqslant 2$ and $t_n\geqslant 2$, then it follows from Lemma 2.5 that

$$\begin{aligned} \frac{1}{\lambda }<m_0\leqslant \frac{d_n}{t_n}\leqslant \frac{d-1}{t_n}\leqslant \frac{d-1}{2}, \end{aligned}$$

which is impossible by Lemma 4.2(i). $\square$

Now we are going to use Theorem 2.15 to prove

### Lemma 4.6

Suppose that $n\geqslant 3$ and *P* is contained in at least two irreducible components of the curve $T_P$ that are different from $T_n$ and that are both smooth at *P*. Then they are tangent to each other at *P*.

### Proof

Without loss of generality, we may assume that $P\in T_1\cap T_2$ and $t_1=t_2=1$. Suppose that $T_1$ and $T_2$ are not tangent to each other at *P*. Put $\Omega =\sum _{i=3}^{n}a_iT_i+\Delta$, so that $D=a_1T_1+a_2T_2+\Omega$. Then $a_1d_1+a_2d_2\leqslant 1$ by Lemma 4.4.

Put $$k_0=\mathrm {mult}(\Omega )$$. Then

$$\begin{aligned} d_1+a_1d_1\big (d-d_1-1\big )-a_2d_1d_2=\Omega \cdot T_1\geqslant k_0 \end{aligned}$$

by Lemma 4.3. Similarly, we have

$$\begin{aligned} d_2-a_1d_1d_2+a_2d_2\big (d-d_2-1\big )=\Omega \cdot T_2\geqslant k_0. \end{aligned}$$

Adding these two inequalities together and using $a_1d_1+a_2d_2\leqslant 1$, we get

$$\begin{aligned} 2k_0\leqslant & {} d_1+d_2+\big (a_1d_1+a_2d_2\big )\big (d-d_1-d_2-1\big )\\\leqslant & {} d_1+d_2+\big (d-d_1-d_2-1\big )=d-1. \end{aligned}$$

Thus, $k_0\leqslant \frac{1}{\lambda }$ by Lemma 4.2(i).

Since $\lambda k_0\leqslant 1$, we can apply Theorem 2.15 to the log pair $(S, \lambda a_1T_1+\lambda a_2T_2+\lambda \Omega )$ at the point *P*. This gives either $\lambda \Omega \cdot T_1>2(1-\lambda a_2)$ or $\lambda \Omega \cdot T_2>2(1-\lambda a_1)$. Without loss of generality, we may assume that $\lambda \Omega \cdot T_2>2(1-\lambda a_1)$. Then

4.1 $$\begin{aligned} d_2+a_2d_2\big (d-d_2-1\big )-a_1d_1d_2=\Omega \cdot T_2>\frac{2}{\lambda }-2a_1. \end{aligned}$$

Applying Theorem 2.12 to the log pair $(S, \lambda a_1T_1+\lambda b_1T_2+\lambda \Omega )$ and the curve $T_1$ at the point *P*, we get

$$\begin{aligned} d_1+a_1d_1\big (d-d_1-1\big )=\Big (\lambda a_2T_2+\lambda \Omega \Big )\cdot T_1>\frac{1}{\lambda }. \end{aligned}$$

Adding this inequality to (4.1), we get

$$\begin{aligned} d+1\geqslant d-1+2a_1\geqslant d_1+d_2+\big (a_1d_1+a_2d_2\big )\big (d-d_1-d_2-1\big )+2a_1>\frac{3}{\lambda }, \end{aligned}$$

because $a_1d_1+a_2d_2\leqslant 1$. Thus, it follows from Lemma 4.2(ii) that either $d=3$ or $d=4$.

If $d=3$, then $n=3$ and $d_1=d_2=d_3=\lambda =1$, which implies that $a_1+a_2>1$ by (4.1). On the other hand, we know that $a_1d_1+a_2d_2\leqslant 1$, so that $a_1+a_2\leqslant 1$. This shows that $d\ne 3$.

We see that $d=4$. Then $\lambda =\frac{5}{8}$ and $d_1+d_2\leqslant 3$. If $d_1=d_1=1$, then (4.1) gives $2a_2+a_1>\frac{11}{5}$. If $d_1=1$ and $d_2=2$, then (4.1) gives $a_2>\frac{3}{5}$. If $d_1=2$ and $d_2=1$, then (4.1) gives $a_2>\frac{11}{5}$. All these three inequalities are inconsistent, because $a_1d_1+a_2d_2\leqslant 1$. The obtained contradiction completes the proof of the lemma. $\square$

Note that every line contained in the surfaces *S* that passes through *P* must be an irreducible component of the curve $T_P$. Moreover, the curve $T_n$ cannot be a line by Lemma 4.5. Thus, Lemma 4.6 implies that there exists at most one line in *S* that passes through *P*. In particular, we see that $n<d$.

### Lemma 4.7

Suppose that $n\geqslant 3$ and *P* is contained in at least two irreducible components of the curve $T_P$ that are different from $T_n$. Then these curves are smooth at *P*.

### Proof

Without loss of generality, we may assume that $P\in T_1\cap T_2$ and $t_1\leqslant t_2$. We have to show that $t_1=t_2=1$. We may assume that $d\geqslant 5$, because the required assertion is obvious in the cases $d=3$ and $d=4$.

Put $\Omega =\sum _{i=3}^{n}a_iT_i+\Delta$ and put $k_0=\mathrm {mult}_{P}(\Omega )$. Then $m_0=k_0+a_1t_1+a_2t_2$. Moreover, we have $a_1d_1+a_2d_2\leqslant 1$ by Lemma 4.4. On the other hand, it follows from Lemma 4.3 that

$$\begin{aligned} d-1 \geqslant d_1+d_2+\big (a_1d_1+a_2d_2\big )\big (d-d_1-d_2-1\big )=\Omega \cdot \Big (T_1+T_2\Big )\geqslant k_0\big (t_1+t_2\big ), \end{aligned}$$

because $a_1d_1+a_2d_2\leqslant 1$. Thus, we have $k_0\leqslant \frac{d-1}{t_1+t_2}$. Hence, if $t_1+t_2\geqslant 4$, then

$$\begin{aligned} m_0=k_0+a_1t_1+a_2t_2\leqslant & {} k_0+a_1d_1+a_2d_2\leqslant \frac{d-1}{t_1+t_2}+a_1d_1+a_2d_2\leqslant \frac{d-1}{t_1+t_2}+1\\\leqslant & {} \frac{d+3}{4} \end{aligned}$$

because $a_1d_1+a_2d_2\leqslant 1$. Since $m_0>\frac{1}{\lambda }$ by Lemma 2.5, the inequality $m_0\leqslant \frac{d+3}{4}$ gives $\lambda >\frac{d+3}{4}$, which is impossible by Lemma 4.2(iii). Thus, $t_1+t_2\leqslant 3$. Since $t_1\leqslant t_2$, we have $t_1=1$ and $t_2\leqslant 2$.

To complete the proof of the lemma, we have to prove that $t_2=1$. Suppose $t_2\ne 1$. Then $t_2=2$, since $t_1+t_2\leqslant 3$. Since $k_0\leqslant \frac{d-1}{t_1+t_2}=\frac{d-1}{3}$ and $a_1d_1+a_2d_2\leqslant 1$, we have

$$\begin{aligned} m_0= & {} k_0+a_1t_1+a_2t_2\leqslant k_0+a_1d_1+a_2d_2\leqslant \frac{d-1}{32}+a_1d_1+a_2d_2\leqslant \frac{d-1}{t_1+t_2}+1\\= & {} \frac{d+2}{3}. \end{aligned}$$

On the other hand, $m_0>\frac{1}{\lambda }$ by Lemma 2.5, so that $\lambda >\frac{3}{d+2}$. Then $d=5$ by Lemma 4.2(iv).

Since $d=5, t_1=1$ and $t_2=2$, we have $n=3, d_1=1, d_2=3$ and $d_3=1$. Applying Theorem 2.12 to the log pair $(S, \lambda a_1T_1+\lambda a_2T_2+\lambda \Omega )$, we get

$$\begin{aligned} 1+3a_1=d_1+a_2d_1\big (d-d_1-1\big )=\Big (\lambda a_2T_2+\lambda \Omega \Big )\cdot T_1>\frac{1}{\lambda }=\frac{15}{7}, \end{aligned}$$

which gives $a_1>\frac{8}{21}$. On the other hand, $a_1+3a_2\leqslant 1$, because $a_1d_1+a_2d_2\leqslant 1$. Since $m_0>\frac{1}{\lambda }=\frac{15}{7}$ by Lemma 2.5, we see that

$$\begin{aligned} \frac{15}{7}-\frac{1}{9}= & {} \frac{128}{63}>\frac{8-5a_1}{3}=\frac{3-a_1+\frac{7(1-a_1)}{3}}{2}=\frac{3-a_1+7a_2}{2}\\= & {} \frac{3-3a_1+3a_2}{2}+a_1+2a_2\\= & {} \frac{\Delta \cdot T_2}{2}+a_1+2a_2\geqslant \frac{\mathrm {mult}_{P}\Big (\Delta \cdot T_2\Big )}{2}+a_1+2a_2\geqslant \frac{t_2k_0}{2}+a_1+2a_2\\= & {} k_0+a_1+2a_2=m_0>\frac{15}{7}, \end{aligned}$$

which is absurd. $\square$

Now we are ready to prove

### Lemma 4.8

One has $$m_0\leqslant \frac{d+1}{2}$$.

### Proof

Suppose that $m_0>\frac{d+1}{2}$. Let us seek for a contradiction. If $n=1$, then

$$\begin{aligned} d=T_n\cdot D\geqslant 2m_0, \end{aligned}$$

which implies that $m_0\leqslant \frac{d}{2}$. Thus, have $n\geqslant 2$. Then $a_1\leqslant \frac{1}{d_1}$ by Lemma 4.4. Moreover, either $t_n=0$ or $t_n=1$ by Lemma 4.5. Hence, there is an irreducible component of $T_P$ that passes through *P* and is different from $T_n$, because $T_P$ is singular at *P*. Without loss of generality, we may assume that $t_1\geqslant 1$.

Put $\Upsilon =\sum _{i=2}^{n}a_iT_i+\Delta$, so that $D=a_1T_1+\Upsilon$. Put $n_0=\mathrm {mult}_{P}(\Upsilon )$, so that $m_0=n_0+a_1t_1$. Then $t_nn_0\leqslant d_n-a_1d_1d_n$ by Lemma 4.4, and

4.2 $$\begin{aligned} d_1+a_1d_1(d-d_1-1)=\Upsilon \cdot T_1\geqslant t_1n_0 \end{aligned}$$

by Lemma 4.3. Adding these two inequalities, we get $(t_1+t_n)n_0\leqslant d_1+d_n+a_1d_1(d-d_1-d_n-1)$. Hence, if $n\geqslant 3$ and $t_n=1$, then

$$\begin{aligned} 2n_0\leqslant \big (t_1+t_n\big )n_0\leqslant d_1+d_n+a_1d_1\big (d-d_1-d_n-1\big )\leqslant d-1\leqslant d-a_1d_1, \end{aligned}$$

because $a_1\leqslant \frac{1}{d_1}$. Similarly, if $n=2$ and $t_n=1$, thenThus, if $t_n=1$, then $n_0\leqslant \frac{d-a_1d_1}{2}$, which is impossible. Indeed, the inequality $n_0\leqslant \frac{d-a_1d_1}{2}$ gives

$$\begin{aligned} \frac{d+1}{2}<m_0=n_0+a_1t_1\leqslant n_0+a_1d_1\leqslant \frac{d-a_1d_1}{2}+a_1d_1=\frac{d+a_1d_1}{2}\leqslant \frac{d+1}{2}, \end{aligned}$$

because $a_1\leqslant \frac{1}{d_1}$. This shows that $t_n=0$.

If $t_1\geqslant 2$, then it follows from (4.2) that

$$\begin{aligned} \frac{d+1}{2}< & {} m_0\leqslant n_0+a_1d_1\leqslant \frac{d_1+a_1d_1(d-d_1-1)}{2}+a_1d_1\\= & {} \frac{d_1+a_1d_1(d-d_1+1)}{2}\leqslant \frac{d+1}{2}, \end{aligned}$$

because $a_1\leqslant \frac{1}{d_1}$. This shows that $t_1=1$.

Since $t_1=1$ and $t_n=0$, there exists an irreducible component of the curve $T_P$ that passes through *P* and is different from $T_1$ and $T_n$. In particular, we have $n\geqslant 3$. Without loss of generality, we may assume $P\in T_2$. Then $T_2$ is smooth at *P* by Lemma 4.7.

Put $\Omega =\sum _{i=3}^{n}a_iT_i+\Delta$ and put $k_0=\mathrm {mult}_{P}(\Omega )$. Then $a_1d_1+a_2d_2\leqslant 1$ by Lemma 4.4. Thus, it follows from Lemma 4.3 that

$$\begin{aligned} 2k_0\leqslant \Omega \cdot \Big (T_1+T_2\Big )=d_1+d_2+\big (a_1d_1+a_2d_2\big )\big (d-d_1-d_2-1\big )\leqslant d-1, \end{aligned}$$

which implies $k_0\leqslant \frac{d-1}{2}$. Then

$$\begin{aligned} \frac{d+1}{2}< & {} m_0=k_0+a_1t_1+a_2t_2\leqslant k_0+a_1d_1+a_2d_2\leqslant \frac{d-1}{2}+a_1d_1+a_2d_2\\\leqslant & {} \frac{d-1}{2}+1=\frac{d+1}{2}, \end{aligned}$$

because $a_1d_1+a_2d_2\leqslant 1$. The obtained contradiction completes the proof of the lemma. $\square$

Let $f_1:S_1\rightarrow S$ be a blow up of the point *P*, and let $E_1$ be its exceptional curve. Denote by $D^1$ the proper transform of the ${\mathbb {Q}}$-divisor *D* on the surface $S_1$. Then

$$\begin{aligned} K_{S_1}+\lambda D^1+\big (\lambda m_0-1\big )E_1\sim _{{\mathbb {Q}}} f_1^{*}\Big (K_{S}+\lambda D\Big ), \end{aligned}$$

which implies that $(S_1, \lambda D^1+(\lambda m_0-1)E_1)$ is not log canonical at some point $P_1\in E_1$.

By Lemma 4.8, we have $m_0\leqslant \frac{d+1}{2}$. By Lemma 4.2(v), we have $\lambda \leqslant \frac{4}{d+1}$. This gives $\lambda m_0\leqslant 2$. Thus, the log pair $(S_1, \lambda D^1+(\lambda m_0-1)E_1)$ is log canonical at every point of the curve $E_1$ that is different from $P_1$ by Lemma 2.13.

Put $m_1=\mathrm {mult}_{P_1}(D^1)$. Then Lemma 2.5 gives

4.3 $$\begin{aligned} m_0+m_1>\frac{2}{\lambda }. \end{aligned}$$

For each curve $T_i$, denote by $T_i^1$ its proper transform on $S_1$. Put $T_P^1=\sum _{i=1}^nT_i^1$.

### Lemma 4.9

One has $$P_1\not \in T_P^1$$.

### Proof

Suppose that $P_1\in T_P^1$. Let us seek for a contradiction. If $T_P$ is irreducible, then

$$\begin{aligned} d-2m_0=T_P^1\cdot D^1\geqslant m_1, \end{aligned}$$

so that $m_1+2m_0\leqslant d$. This inequality gives

$$\begin{aligned} \frac{3}{\lambda }<m_1+2m_0\leqslant d, \end{aligned}$$

because $2m_0\geqslant m_0+m_1>\frac{2}{\lambda }$ by (4.3). This shows that $T_P$ is reducible, because $\lambda \leqslant \frac{3}{d}$ by Lemma 4.2(vi).

We see that $n\geqslant 2$. If $P_1\in T_n^1$, then

$$\begin{aligned} d-1-m_0\geqslant d_n-m_0=d_n-m_0t_n=T_n^1\cdot D^1\geqslant m_1, \end{aligned}$$

which is impossible, because $m_0+m_1>\frac{2}{\lambda }$ by (4.3), and $\lambda \leqslant \frac{2}{d-1}$ by Lemma 4.2(i). Thus, we see that $P_1\not \in T_n^1$.

Without loss of generality, we may assume that $P_1\in T_1^1$. Put $\Upsilon =\sum _{i=2}^{n}a_iT_i+\Delta$, and denote by $\Upsilon ^1$ the proper transform of the ${\mathbb {Q}}$-divisor $\Omega$ on the surface $S_1$. Put $n_0=\mathrm {mult}_{P}(\Upsilon )$, put $n_1=\mathrm {mult}_{P_1}(\Omega ^1)$ and put $t_1^1=\mathrm {mult}_{P_1}(T_1^1)$. Then

$$\begin{aligned} d_1+a_1d_1\big (d-d_1-1\big )-n_0t_1=T_1^1\cdot \Upsilon ^1\geqslant t_1^1n_1, \end{aligned}$$

which implies that $n_0t_1+n_1t_1^1\leqslant d_1+a_1d_1(d-d_1-1)$.

Note that $t_1^1\leqslant t_1$. Moreover, we have $a_1\leqslant \frac{1}{d_1}$ by Lemma 4.4. Thus, if $t_1^1\geqslant 2$, then

$$\begin{aligned} 2\big (n_0+n_1\big )\leqslant & {} t_1^1\big (n_0+n_1\big )\leqslant n_0t_1+n_1t_1^1\leqslant d_1+a_1d_1\big (d-d_1-1\big )\\\leqslant & {} d_1+\big (d-d_1-1\big )=d-1, \end{aligned}$$

which implies that $n_0+n_1\leqslant \frac{d-1}{2}$. Moreover, if $n_0+n_1\leqslant \frac{d-1}{2}$, then it follows from (4.3) that

$$\begin{aligned} \frac{d+3}{2}= & {} 2+\frac{d-1}{2}\geqslant 2a_1d_1+\frac{d-1}{2}\geqslant 2a_1t_1+\frac{d-1}{2}\geqslant a_1\big (t_1+t_1^1\big )+n_0+n_1\\= & {} m_0+m_1>\frac{2}{\lambda } \end{aligned}$$

which gives $d\leqslant 4$ by Lemma 4.2(iii). Thus, if $d\geqslant 5$, then $t_1^1=1$. Furthermore, if $d\leqslant 4$, then $d_1\leqslant 3$, which implies that $t_{1}^1\leqslant 1$. This shows that $t_1^1=1$ in all cases. Thus, the curve $T_1^1$ is smooth at $P_1$.

Applying Theorem 2.11 to the log pair $(S_1, \lambda \Upsilon ^1+\lambda a_1T_1^1+(\lambda (n_0+a_1t_1)-1)E_1)$, we see that

$$\begin{aligned} \lambda \big (d-1-n_0t_1\big )\geqslant & {} \lambda \Big (d_1+a_1d_1\big (d-d_1-1\big )-n_0t_1\Big )\\= & {} \lambda \Omega ^1\cdot T_1^1>2-\lambda \big (n_0+a_1t_1\big ), \end{aligned}$$

because $a_1\leqslant \frac{1}{d_1}$. Thus, we have $d-1+a_1t_1-n_0(t_1-1)>\frac{2}{\lambda }$. But $m_0=a_1t_1+n_0>\frac{1}{\lambda }$ by Lemma 2.5. Adding these inequalities together, we obtain

4.4 $$\begin{aligned} d-1+2a_1t_1-n_0(t_1-2)>\frac{3}{\lambda }. \end{aligned}$$

If $t_1\geqslant 2$, this gives

$$\begin{aligned} d+1\geqslant d-1+2a_1d_1\geqslant d-1+2a_1t_1\geqslant d-1+2a_1t_1-n_0(t_1-2)>\frac{3}{\lambda }. \end{aligned}$$

because $a_1\leqslant \frac{1}{d_1}$. One the other hand, if $d\geqslant 5$, then $\lambda \leqslant \frac{3}{d+1}$ by Lemma 4.2(ii). Thus, if $d\geqslant 5$, then $t_1=1$. Moreover, if $d=3$, then $d_1\leqslant 2$, which implies that $t_1=1$ as well. Furthermore, if $d=4$ and $t_1\ne 1$, then $d_1=3, t_1=2, \lambda =\frac{5}{8}$, which implies that

$$\begin{aligned} \frac{1}{3}=\frac{1}{d_1}\geqslant a_1>\frac{9}{20} \end{aligned}$$

by (4.4). Thus, we see that $t_1=1$ in all cases. This simply means that the curve $T_1$ is smooth at the point *P*.

Since $a_1\leqslant \frac{1}{d_1}$, we have

$$\begin{aligned} d-1-n_0\geqslant d_1+a_1d_1\big (d-d_1-1\big )-n_0=\Omega ^1\cdot T_1^1\geqslant n_1, \end{aligned}$$

which implies that $n_1\leqslant \frac{n_0+n_1}{2}\leqslant \frac{d-1}{2}$. Then $\lambda n_1\leqslant 1$ by Lemma 4.2(i). Hence, we can apply Theorem 2.15 to the log pair $(S_1, \lambda \Upsilon ^1+\lambda a_1T_1^1+(\lambda (n_0+a_1t_1)-1)E_1)$ at the point $P_1$. This gives either

$$\begin{aligned} \Upsilon ^1\cdot T_1^1>\frac{4}{\lambda }-2(n_0+a_1) \end{aligned}$$

or $\Upsilon ^1\cdot E_1>\frac{2}{\lambda }-2a_1$ (or both). Since $a_1\leqslant \frac{1}{d_1}$, the former inequality gives

$$\begin{aligned} d-1-n_0\geqslant d_1+a_1d_1\big (d-d_1-1\big )-n_0=\Upsilon ^1\cdot T_1^1>\frac{4}{\lambda }-2(n_0+a_1). \end{aligned}$$

Similarly, the latter inequality gives

$$\begin{aligned} n_0=\lambda \Upsilon ^1\cdot E_1>\frac{2}{\lambda }-2a_1. \end{aligned}$$

Thus, either $d-1+2a_1+n_0>\frac{4}{\lambda }$ or $2a_1+n_0>\frac{2}{\lambda }$ (or both).

If $t_n\geqslant 1$, then $d_n\ne 1$ by Lemma 4.5. Thus, if $t_n\geqslant 1$, then

$$\begin{aligned} d-1\geqslant d_n\geqslant a_1d_1d_n+n_0\geqslant 2a_1+n_0 \end{aligned}$$

by Lemma 4.4. Therefore, if $t_n\geqslant 1$, then

$$\begin{aligned} 2(d-1)\geqslant d-1+2a+n_0>\frac{4}{\lambda } \end{aligned}$$

or $d-1\geqslant 2a+n_0>\frac{2}{\lambda }$, because $d-1+2a+n_0>\frac{4}{\lambda }$ or $2a+n_0>\frac{2}{\lambda }$. In both cases, we get $\lambda >\frac{d-1}{2}$, which is impossible by Lemma 4.2(i). This shows that $t_n=0$, so that $P\not \in T_n$.

Since $T_1$ is smooth at *P* and $P\not \in T_n$, there must be another irreducible component of $T_P$ passing through *P* that is different from $T_1$ and $T_n$. In particular, we see that $n\geqslant 3$. Without loss of generality, we may assume that $P\in T_2$. Then $T_2$ is smooth at *P* by Lemma 4.7, so that $t_2=1$. Moreover, the curves $T_1$ and $T_2$ are tangent at *P* by Lemma 4.6, which implies that $d\geqslant 4$. Since $P_1\in T_1^1$, we see that $P_1\in T_2^1$ as well.

Put $\Omega =\sum _{i=3}^{n}a_iT_i+\Delta$ and $k_0=\mathrm {mult}_{P}(\Omega )$, so that $m_0=k_0+a_1+a_2$. Then $a_1d_1+a_2d_2\leqslant 1$ by Lemma 4.4.

Denote by $\Omega ^1$ the proper transform of the ${\mathbb {Q}}$-divisor $\Omega$ on the surface $S_1$. Put $k_1=\mathrm {mult}_{P_1}(\Omega ^1)$. Then

$$\begin{aligned} d-1-2k_0\geqslant & {} d_1+d_2+\big (a_1d_1+a_2d_2\big )\big (d-d_1-d_2-1\big )-2k_0\\= & {} \Omega ^1\cdot \Big (T_1^1+T_2^1\Big )\geqslant 2k_1 \end{aligned}$$

because $a_1d_1+a_2d_2\leqslant 1$ and $d\geqslant d_1+d_2+d_n\geqslant d_1+d_2+1$. This gives $k_0+k_1\leqslant \frac{d-1}{2}$. On the other hand, we have

$$\begin{aligned} 2a_1+2a_2+k_0+k_1=m_0+m_1>\frac{2}{\lambda } \end{aligned}$$

by (4.3). Thus, we have

$$\begin{aligned} \frac{d+3}{2}= & {} 2+\frac{d-1}{2}\geqslant 2\big (a_1d_1+a_2d_2\big )+\frac{d-1}{2}\geqslant 2a_1+2a_2+\frac{d-1}{2}\\\geqslant & {} 2a_1+2a_2+k_0+k_1>\frac{2}{\lambda } \end{aligned}$$

because $a_1d_1+a_2d_2\leqslant 1$. By Lemma 4.2(iii) this gives $d=4$. Thus, we have $\lambda =\frac{5}{8}$.

Since $d=4>n\geqslant 3$, we have $n=3$. Without loss of generality, we may assume that $d_1\leqslant d_2$. By Lemma 4.6, there exists at most one line in *S* that passes through *P*. This shows that $d_1=1, d_2=2$ and $d_3=1$. Thus, $T_1$ and $T_3$ are lines, $T_2$ is a conic, $T_1$ is tangent to $T_2$ at *P*, and $T_3$ does not pass through *P*. In particular, the curves $T_1^1$ and $T_1^2$ intersect each other transversally at $P_1$.

By Lemma 4.3, we have $T_1\cdot T_1=T_2\cdot T_2=-2$ and $T_1\cdot T_2=2$. On the other hand, the log pair $(S_1, \lambda a_1 T_1^1+\lambda a_2 T_2^1+\lambda \Omega ^1+(\lambda (a_1+a_2+k_0)-1)E_1)$ is not log canonical at the point $P_1$. Thus, applying Theorem 2.11 to this log pair and the curve $T_1^1$, we get

$$\begin{aligned} \lambda \big (1+2a_1-2a_2-k_0\big )=\lambda \Omega ^1\cdot T_1^1>2-\lambda (a_1+a_2+k_0)-\lambda a_2, \end{aligned}$$

which implies that $3a_1>\frac{2}{\lambda }-1=\frac{11}{5}$, because $\lambda =\frac{5}{8}$. Similarly, applying Theorem 2.11 to this log pair and the curve $T_2^1$, we get

$$\begin{aligned} \lambda \big (2-2a_1+2a_2-k_0\big )=\lambda \Omega ^1\cdot T_2^1>2-\lambda (a_1+a_2+k_0)-\lambda a_1, \end{aligned}$$

which implies that $3a_2>\frac{2}{\lambda }-2=\frac{6}{5}$. Hence, we have $a_1>\frac{11}{15}$ and $a_2>\frac{2}{5}$, which is impossible, since $a_1+2a_2=a_1d_1+a_2d_2\leqslant 1$. The obtained contradiction completes the proof of the lemma. $\square$

Now we are going to show that the curve $T_P$ has at most two irreducible components. This follows from

### Lemma 4.10

One has $n\geqslant 2$ and $\mathrm {mult}_{P}(T_P)=2$. Moreover, if $n=2$, then $P\in T_1\cap T_2$, both curves $T_1$ and $T_2$ are smooth at *P*, and $d_1\leqslant d_2$.

### Proof

If $T_P$ is irreducible and $\mathrm {mult}_{P}(T_P)\geqslant 3$, then Lemma 2.5 gives

$$\begin{aligned} d=T_P\cdot D\geqslant 3m_0>\frac{3}{\lambda }, \end{aligned}$$

which is impossible by Lemma 4.2(vi). Thus, if $n=1$, then $\mathrm {mult}_{P}(T_P)=2$.

To complete the proof, we may assume that $n\geqslant 2$. Then $t_n=0$ or $t_n=1$ by Lemma 4.5. In particular, there exists an irreducible component of the curve $T_P$ different from $T_n$ that passes through *P*. Without loss of generality, we may assume that $P\in T_1$.

Put $\Upsilon =\sum _{i=2}^{n}a_iT_i+\Delta$, and denote by $\Upsilon ^1$ the proper transform of the ${\mathbb {Q}}$-divisor $\Omega$ on the surface $S_1$. Put $n_0=\mathrm {mult}_{P}(\Upsilon )$. Then the log pair $(S_1, \lambda \Upsilon ^1+(\lambda (n_0+a_1t_1)-1)E_1)$ is not log canonical at $P_1$, since $P_1\not \in T_1^1$ by Lemma 4.9. In particular, it follows from Theorem 2.12 that

$$\begin{aligned} \lambda n_0=\lambda \Upsilon ^1\cdot E_1>1, \end{aligned}$$

which implies that $n_0>\frac{1}{\lambda }$. Thus, if $t_1\geqslant 2$, then it follows from Lemma 4.3 that

$$\begin{aligned} \frac{1}{\lambda }\geqslant \frac{d-1}{2}\geqslant \frac{d_1+a_1d_1(d-d_1-1)}{2}=\frac{\Upsilon \cdot T_1}{2}\geqslant \frac{t_1n_0}{2}\geqslant n_0>\frac{1}{\lambda }, \end{aligned}$$

because $a_1\leqslant \frac{1}{d_1}$ by Lemma 4.4, and $\lambda \leqslant \frac{2}{d-1}$ by Lemma 4.2(i). This shows that $t_1=1$, so that the curve $T_1$ is smooth at *P*.

If $t_n=1$ and $n\geqslant 3$, then

$$\begin{aligned} \frac{2}{\lambda }\geqslant d-1\geqslant d_1+d_n+ad_1(d-d_1-d_n-1)=\Upsilon \cdot \Big (T_1+T_n\Big )\geqslant 2n_0>\frac{2}{\lambda }. \end{aligned}$$

Thus, if $t_n=1$, then $n=2$. Vice versa, if $n=2$, then $t_n=1$, because $T_1$ is smooth at *P*. Furthermore, if $n=2$, then $d_1\leqslant d_n$, because $d_n>\frac{d-1}{2}$ by Lemma 4.5. Therefore, to complete the proof, we must show that $n=2$.

Suppose that $n\geqslant 3$. Let us seek for a contradiction. We know that $P\not \in T_n$, so that $t_n=0$. Then every irreducible component of the curve $T_P$ that contain *P* is smooth at *P* by Lemma 4.7. Hence, there should be at least one irreducible component of the curve $T_P$ containing *P* that is different from $T_1$ and $T_n$. Without loss of generality, we may assume that $P\in T_2$.

Put $\Omega =\sum _{i=3}^{n}a_iT_i+\Delta$ and $k_0=\mathrm {mult}_{P}(\Omega )$. By Lemma 4.4, we have $a_1d_1+a_2d_2\leqslant 1$. Thus, it follows from Lemma 4.3 that

$$\begin{aligned} 2k_0\leqslant & {} \Omega \cdot \Big (T_1+T_2\Big )=d_1+d_2+\big (a_1d_1+a_2d_2\big )\big (d-d_1-d_2-1\big )\\\leqslant & {} d_1+d_2+\big (d-d_1-d_2-1\big )=d-1. \end{aligned}$$

Hence, we have $k_0\leqslant \frac{d-1}{2}$.

Denote by $\Omega ^1$ the proper transform of the ${\mathbb {Q}}$-divisor $\Omega$ on the surface $S_1$. Then the log pair $(S_1, \lambda \Omega ^1+(\lambda (k_0+a_1+a_2)-1)E_1)$ is not log canonical at $P_1$, because $P_1\not \in T_1^1$ and $P_1\not \in T_2^1$ by Lemma 4.9. In particular, it follows from Theorem 2.11 that

$$\begin{aligned} \lambda k_0=\lambda \Omega ^1\cdot E_1>1, \end{aligned}$$

which implies that $k_0>\frac{1}{\lambda }$. This contradicts Lemma 4.2(i), because $k_0\leqslant \frac{d-1}{2}$. $\square$

Later, we will need the following simple

### Lemma 4.11

Suppose that $d=4$. Then $m_0\leqslant \frac{11}{5}$.

### Proof

If $$n=1$$, then

$$\begin{aligned} 2t_n\geqslant d_n=T_n\cdot D\geqslant t_nm_0, \end{aligned}$$

so that $m_0\leqslant 2<\frac{11}{5}$. Thus, we may assume that $n\ne 1$. Then it follows from Lemma 4.10 that $n=2, P\in T_1\cap T_2$, both curves $T_1$ and $T_2$ are smooth at *P*, and $d_1\leqslant d_2$.

If $d_2=2$, then $m_0\leqslant 2<\frac{11}{5}$, because

$$\begin{aligned} 2=T_2\cdot D\geqslant m_0. \end{aligned}$$

Thus, we may assume that $d_2\ne 2$. Then $d_1=1$ and $d_2=3$. Then $\mathrm {mult}_{P}(\Delta )+3a_1\leqslant 3$ by Lemma 4.4. Moreover, we have

$$\begin{aligned} 1+2a_1=T_1\cdot \Delta \geqslant \mathrm {mult}_{P}(\Delta ). \end{aligned}$$

The obtained inequalities give $m_0=\mathrm {mult}_{P}(\Delta )+a_1\leqslant \frac{11}{5}$. $\square$

Let $f_2:S_2\rightarrow S_1$ be a blow up of the point $P_1$. Denote by $E_2$ the $f_2$-exceptional curve, denote by $E_1^2$ the proper transform of the curve $E_1$ on the surface $S_2$, and denote by $D^2$ the proper transform of the ${\mathbb {Q}}$-divisor *D* on the surface $S_2$. Then

$$\begin{aligned}&K_{S_2}+\lambda D^2+\big (\lambda m_0-1\big )E_1^2+\Big (\lambda \big (m_0+m_1\big )-2\Big )E_2\sim _{{\mathbb {Q}}} f_2^*\Big (K_{S_1}+\lambda D^1\\&\quad +\,\big (\lambda m_0-1\big )E_1\Big ). \end{aligned}$$

By Remark 2.10, the log pair $(S_2, \lambda D^2+(\lambda m_0-1)E_1^2+(\lambda (m_0+m_1)-2)E_2)$ is not log canonical at some point $P_2\in E_1$.

### Lemma 4.12

One has $$m_0+m_1\leqslant \frac{3}{\lambda }$$.

### Proof

Suppose that $m_0+m_1>\frac{3}{\lambda }$. Then $2m_0\geqslant m_0+m_1>\frac{3}{\lambda }$. But $m_0\leqslant \frac{d+1}{2}$ by Lemma 4.8. Then $\lambda >\frac{3}{d+1}$. Thus, we have $d\leqslant 4$ by Lemma 4.2(ii). Moreover, if $d=4$, then

$$\begin{aligned} \frac{22}{5}\geqslant 2m_0\geqslant m_0+m_1>\frac{3}{\lambda }=\frac{24}{5} \end{aligned}$$

by Lemma 4.11. This shows that $d=3$.

We have $\lambda =1$. If $n=1$, then

$$\begin{aligned} 3=T_P\cdot D\geqslant 2m_0\geqslant m_1+m_0>\frac{3}{\lambda }=3, \end{aligned}$$

which is absurd. Hence, it follows from Lemma 4.10 that $n=2, d_1=1, d_2=2$ and $P\in T_1\cap T_2$.

We have $m_0=\mathrm {mult}_{P}(\Delta )+a_1$. On the other hand, we have $\mathrm {mult}_{P}(\Delta )+2a_1\leqslant 2$ by Lemma 4.4. Moreover, we have

$$\begin{aligned} 1+a_1=T_1\cdot \Omega \geqslant \mathrm {mult}_{P}(\Delta ), \end{aligned}$$

which implies that $\mathrm {mult}_{P}(\Delta )-a_1\leqslant 1$. Adding these inequalities, we get

$$\begin{aligned} 3\geqslant 2\mathrm {mult}_{P}(\Delta )+a=\mathrm {mult}_{P}(\Delta )+m_0\geqslant m_1+m_0>\frac{3}{\lambda }=3, \end{aligned}$$

because $\mathrm {mult}_{P}(\Delta )\geqslant m_1$, since $P_1\not \in T_1^1$ by Lemma 4.9. $\square$

Thus, the log pair $(S_2, \lambda D^2+(\lambda m_0-1)E_1^2+(\lambda (m_0+m_1)-2)E_2)$ is log canonical at every point of the curve $E_2$ that is different from the point *P* by Lemma 2.13.

### Lemma 4.13

One has $$P_2\ne E_1^2\cap E_2$$.

### Proof

Suppose that $P_2=E_1^2\cap E_2$. Then Theorem 2.11 gives

$$\begin{aligned} \lambda \big (m_0-m_1\big )=\lambda D^2\cdot E_1^2>3-\lambda \big (m_0+m_1\big ), \end{aligned}$$

which implies that $m_0>\frac{3}{2\lambda }$. But $m_0\leqslant \frac{d+1}{2}$ by Lemma 4.8. Therefore, we have $\lambda >\frac{3}{d+1}$, which implies that $d\leqslant 4$ by Lemma 4.2(ii). If $d=4$, then

$$\begin{aligned} \frac{12}{5}=\frac{3}{2\lambda }<m_0\leqslant \frac{11}{5} \end{aligned}$$

by Lemma 4.11. Thus, we have $d=3$.

One has $\lambda =1$. If $n=1$, then

$$\begin{aligned} 3=T_P\cdot D\geqslant 2m_0>\frac{3}{\lambda }=3, \end{aligned}$$

which is absurd. Hence, it follows from Lemma 4.10 that $n=2, d_1=1, d_2=2$ and $P\in T_1\cap T_2$.

We have $m_0=\mathrm {mult}_{P}(\Delta )+a_1$. Moreover, we have $\mathrm {mult}_{P}(\Delta )+2a_1\leqslant 2$ by Lemma 4.4, Then $2\mathrm {mult}_{P}(\Delta )+a_1\leqslant 3$, because

$$\begin{aligned} 1+a_1=T_1\cdot \Delta \geqslant \mathrm {mult}_{P}(\Delta ). \end{aligned}$$

Denote by $\Delta ^1$ the proper transform of the divisor $\Delta$ on the surface $S_1$, and denote by $\Delta ^2$ the proper transform of the divisor $\Delta$ on the surface $S_2$. Then $m_1=\mathrm {mult}_{P_1}(\Delta ^1)$, because $P_1\not \in T_1^1$ by Lemma 4.9. Thus, the log pair $(S_2, \lambda \Delta ^2+(m_0-1)E_1^2+(m_0+m_1-2)E_2)$ is not log canonical at $P_2$. Applying Theorem 2.11 to this pair and the curve $E_1^2$, we get

$$\begin{aligned} \mathrm {mult}_{P}(\Delta )-m_1=\Delta ^2\cdot E_1^2>3-m_0-m_1, \end{aligned}$$

which implies that $2\mathrm {mult}_{P}(\Delta )+a_1>3$. The latter is impossible, because we already proved that $2\mathrm {mult}_{P}(\Delta )+a_1\leqslant 3$. $\square$

Thus, the log pair $(S_2, \lambda D^2+(\lambda (m_0+m_1)-2)E_2)$ is not log canonical at $P_2$. Then Lemma 2.5 gives

4.5 $$\begin{aligned} m_0+m_1+m_2>\frac{3}{\lambda }. \end{aligned}$$

Denote by $T_P^2$ the proper transform of the curve $T_P$ on the surface $S^2$. Then

$$\begin{aligned} T_P^2+E_1^2\sim (f_{1}\circ f_2)^*({\mathcal {O}}_{S}(1))-f_2^*(E_1)-E_2, \end{aligned}$$

because $T_P^1\sim f_{1}^*({\mathcal {O}}_{S}(1))-2E_1$ by Lemma 4.10, and $P_1\not \in T_P^1$ by Lemma 4.9.

### Lemma 4.14

The linear system $|T_P^2+E_1^2|$ is a pencil that does not have base points in $E_2$.

### Proof

Since $|T_P^1+E_1|$ is a two-dimensional linear system that does not have base points, $|T_P^2+E_1^2|$ is a pencil. Let *C* be a curve in $|T_P^1+E_1|$ that passes through $P_1$ and is different from $T_P^1+E_1$. Then *C* is smooth at *P*, since $P\in f_1(C)$ and $f_1(C)$ is a hyperplane section of the surface *S* that is different from $T_P$. Since $C\cdot E_1=1$, we see that $T_P^1+E_1$ and *C* intersect transversally at $P_1$. Thus, the proper transform of the curve *C* on the surface $S_2$ is contained in $|T_P^1+E_1|$ and have no common points with $T_P^2+E_1^2$ in $E_2$. This shows that the pencil $|T_P^1+E_1|$ does not have base points in $E_2$. $\square$

Let $Z^2$ be the curve in $|T_P^2+E_2|$ that passes through the point $P_2$. Then

$$\begin{aligned} Z^2\ne T_P^2+E_1^2, \end{aligned}$$

because $P_2\ne E_1^2\cap E_2$ by Lemma 4.13. Then $Z_2$ is smooth at $P_2$. Put $Z=f_1\circ f_2(Z^2)$ and $Z^1=f_2(Z^2)$. Then $P\in Z$ and $P_1\in Z^1$. Moreover, the curve *Z* is smooth at *P*, and the curve $Z_1$ is smooth at $P_1$. Furthermore, the curve *Z* is reduced by Lemma 2.6.

The log pair $(S,\lambda Z)$ is log canonical at *P*, because *Z* is smooth at *P*. Note that

$$\begin{aligned} Z\sim _{{\mathbb {Q}}} D. \end{aligned}$$

Thus, we may assume that $\mathrm {Supp}(D)$ does not contain at least one irreducible component of the curve *Z* by Remark 2.4. Denote this irreducible component by ${\overline{Z}}$, and denote its degree in ${\mathbb {P}}^3$ by ${\bar{d}}$. Then ${\bar{d}}\leqslant d$.

### Lemma 4.15

One has $$P\not \in {\overline{Z}}$$.

### Proof

Suppose that $P\in {\overline{Z}}$. Let us seek for a contradiction. Denote by ${\overline{Z}}^2$ the proper transform of the curve ${\overline{Z}}$ on the surface $S_2$. Then

$$\begin{aligned} d-m_0-m_1\geqslant {\bar{d}}-m_0-m_1={\overline{Z}}^2\cdot D^2\geqslant m_2, \end{aligned}$$

which implies that $m_0+m_1+m_2\leqslant d$. One the other hand, $m_0+m_1+m_2>\frac{3}{\lambda }$ by (4.5). This gives $\lambda >\frac{3}{d}$, which is impossible by Lemma 4.2(vi). $\square$

In particular, the curve *Z* is reducible. Denote by ${\widehat{Z}}$ its irreducible component that passes through *P*, denote its proper transform on the surface $S_1$ by ${\widehat{Z}}^1$, and denote its proper transform on the surface $S_2$ by ${\widehat{Z}}^2$. Then ${\overline{Z}}\ne {\widehat{Z}}, P_1\in {\widehat{Z}}^1$ and $P_2\in {\widehat{Z}}^2$. Denote by ${\hat{d}}$ the degree of the curve ${\widehat{Z}}$ in ${\mathbb {P}}^3$. Then ${\hat{d}}+{\bar{d}}\leqslant d$. Moreover, the intersection form of the curves ${\widehat{Z}}$ and ${\overline{Z}}$ on the surface *S* is given by

### Lemma 4.16

One has ${\overline{Z}}\cdot {\overline{Z}}=-{\bar{d}}(d-{\bar{d}}-1), {\widehat{Z}}\cdot {\widehat{Z}}=-{\hat{d}}(d-{\hat{d}}-1)$ and ${\overline{Z}}\cdot {\widehat{Z}}={\bar{d}}{\hat{d}}$.

### Proof

See the proof of Lemma 4.3. $\square$

Put $D=a{\widehat{Z}}+\Omega$, where *a* is a positive rational number, and $\Omega$ is an effective ${\mathbb {Q}}$-divisor on the surface *S* whose support does not contain the curve ${\widehat{Z}}$. Denote by $\Omega ^1$ the proper transform of the divisor $\Omega$ on the surface $S_1$, and denote by $\Omega ^2$ the proper transform of the divisor $\Omega$ on the surface $S_2$. Put $n_0=\mathrm {mult}_{P}(\Omega ), n_1=\mathrm {mult}_{P_1}(\Omega ^1)$ and $n_2=\mathrm {mult}_{P_2}(\Omega ^2)$. Then $m_0=n_0+a, m_1=n_1+a$ and $m_2=n_2+a$. Then the log pair $(S_2, \lambda a{\widehat{Z}}^2+\lambda \Omega ^2+(\lambda (n_0+n_1+2a)-2)E_2)$ is not log canonical at $P_2$, because $(S_2, \lambda D^2+(\lambda (m_0+m_1)-2)E_2)$ is not log canonical at $P_2$. Thus, applying Theorem 2.11, we see that

$$\begin{aligned} \lambda \Big (\Omega \cdot {\widehat{Z}}-n_0-n_1\Big )= & {} \lambda \Omega ^2\cdot Z^2>1-\Big (\lambda \big (n_0+n_1+2a\big )-2\Big )\\= & {} 3-\lambda \big (n_0+n_1+2a\big ), \end{aligned}$$

which implies that

4.6 $$\begin{aligned} \Omega \cdot {\widehat{Z}}>\frac{3}{\lambda }-2a. \end{aligned}$$

On the other hand, we have

$$\begin{aligned} {\bar{d}}=D\cdot {\overline{Z}}=\Big (a{\widehat{Z}}+\Omega \Big )\cdot {\overline{Z}}\geqslant a{\widehat{Z}}\cdot {\overline{Z}}=a{\hat{d}}{\bar{d}} \end{aligned}$$

by Lemma 4.16. This gives

4.7 $$\begin{aligned} a\leqslant \frac{1}{{\hat{d}}}. \end{aligned}$$

Thus, it follows from (4.6), (4.7) and Lemma 4.16 that

$$\begin{aligned} \frac{3}{\lambda }-2\leqslant \frac{3}{\lambda }-2a<\Omega \cdot {\widehat{Z}}={\hat{d}}+a{\hat{d}}\Big (d-{\hat{d}}-1\Big )\leqslant d-1, \end{aligned}$$

which implies that $\lambda >\frac{3}{d+1}$. Then $d\leqslant 4$ by Lemma 4.2(ii).

### Lemma 4.17

One has $$d\ne 4$$.

### Proof

Suppose that $d=4$. Then $\lambda =\frac{5}{8}$ and ${\hat{d}}\leqslant 3$. By Lemma 4.9, ${\widehat{Z}}$ is not a line, since every line passing through *P* must be an irreducible component of the curve $T_P$. Thus, either ${\widehat{Z}}$ is a conic or ${\widehat{Z}}$ is a plane cubic curve. If ${\widehat{Z}}$ is a conic, then ${\widehat{Z}}^2=-2$ and $a\leqslant \frac{1}{2}$ by (4.7). Thus, if ${\widehat{Z}}$ is a conic, then

$$\begin{aligned} 2+2a=\Omega \cdot {\widehat{Z}}>\frac{3}{\lambda }-2a=\frac{24}{5}-2a, \end{aligned}$$

which implies that $\frac{1}{2}\geqslant a>\frac{7}{10}$. This shows that ${\widehat{Z}}$ is a plane cubic curve. Then ${\widehat{Z}}^2=0$. Since $a\leqslant \frac{1}{3}$ by (4.7), we have

$$\begin{aligned} 3=\Omega \cdot {\widehat{Z}}>\frac{3}{\lambda }-2a=\frac{24}{5}-2a\geqslant \frac{24}{5}-\frac{2}{3}=\frac{62}{15}, \end{aligned}$$

which is absurd. $\square$

Thus, we see that $d=3$. Then ${\widehat{Z}}$ is either a line or a conic. But every line passing through *P* must be an irreducible component of $T_P$. Since ${\widehat{Z}}$ is not an irreducible component of $T_P$ by Lemma 4.9, the curve ${\widehat{Z}}$ must be a conic. Then ${\widehat{Z}}^2=0$. Therefore, it follows from (4.6) that

$$\begin{aligned} 3-2a=\frac{3}{\lambda }-2a<\Omega \cdot {\widehat{Z}}={\hat{d}}+a{\hat{d}}\Big (d-{\hat{d}}-1\Big )={\hat{d}}=2, \end{aligned}$$

which implies that $a>\frac{1}{2}$. But $a\leqslant \frac{1}{{\hat{d}}}=\frac{1}{2}$ by (4.7). The obtained contradiction completes the proof of Theorem 1.17.
